# Ligand-Controlled Valley and Spin Properties in Ni-Based Two-Dimensional Metal–Organic Frameworks

**DOI:** 10.1021/acs.jpcc.6c01839

**Published:** 2026-07-04

**Authors:** Nafiseh Falsafi, Saeed H. Abedinpour, Fariba Nazari, Francesc Illas

**Affiliations:** 1 Department of Chemistry, Institute for Advanced Studies in Basic Sciences, Zanjan 45137-66731, Iran; 2 Department of Physics, Institute for Advanced Studies in Basic Sciences, Zanjan 45137-66731, Iran; 3 Center of Climate Change and Global Warming, Institute for Advanced Studies in Basic Sciences, Zanjan 45137-66731, Iran; 4 Departament de Ciència de Materials i Química Física & Institut de Química Teòrica i Computacional (IQTCUB), 16724 Universitat de Barcelona,C/Martí i Franquès 1, Barcelona 08028, Spain

## Abstract

First-principles calculations based on density functional theory are employed to investigate the interplay among charge, orbital, lattice, valley, and porosity degrees of freedom in ligand-substituted two-dimensional metal–organic frameworks, Ni_3_C_12_X_12_ (X = O, S, and Se). Depending on the ligand configuration and the level of substitution (half or full), the structures fall into three groups: *cis*-like, *trans*-like, and homogeneous. Modulating the charge degrees of freedom shifts the Fermi level into spin–orbit coupling gaps, enabling the emergence of nontrivial topological features across most of the families. In *cis*-like structures, broken space inversion symmetry simultaneously tunes the lattice and orbital degrees of freedom, opening a valley Hall gap accompanied by spin splitting. The magnitude of this spin splitting scales with the inner potential differences induced by ligands of varying electronegativity. This potential difference also reshapes the Berry curvature so that larger potential contrasts suppress its peak value while broadening its distribution across the Brillouin zone. *Trans*-like configurations with broken mirror symmetry, preserve nontrivial topological characteristics—including quantized spin Hall conductivity (in most cases), a finite Z_2_ invariant, and helical edge states—under low electron doping. Nevertheless, *trans*-like structures with O-ligand can undergo a transition to a Z_2_ metallic phase at higher electron doping concentrations, driven by band gap closure. Homogeneous structures maintain their two-dimensional topological-insulator character except in two cases with large tensile tension.

## Graphical abstract

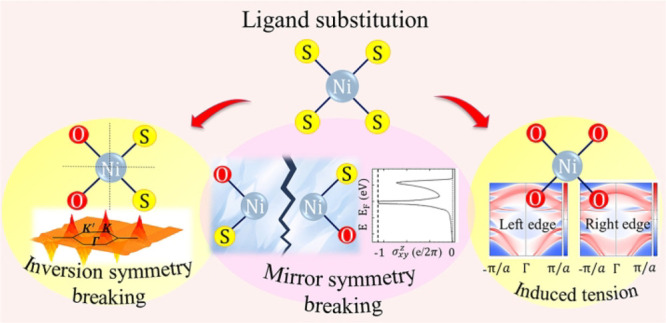

## 1 Introduction

A well-known feature of the spin Hall effect (SHE)^1^ is that it enables the conversion between spin– and charge– degrees of freedom (DOFs). Indeed, efficient charge-to-spin conversion is crucial for spin-based devices of interest in spintronics. Therefore, charge-to-spin conversion control, has attracted considerable attention in condensed matter physics, and manipulating the symmetry of a system has been proposed as an effective route.^2^ Achieving such symmetry-driven control, however, requires materials that exhibit specific and often unconventional characteristics, including well-defined structural, electronic, or dynamical asymmetries that can be deliberately tuned. The identification and design of materials possessing these attributes are therefore central to realizing practical control of charge-to-spin conversion. In this context, metal–organic frameworks (MOFs) have emerged as one of the most promising platforms, owing to their exceptional design flexibility. This flexibility allows precise tuning of pore architecture, chemical functionality, and structural adaptability at the molecular level.

Unlike conventional quantum materials, MOFs host multiple unconventional DOFs, providing opportunities to realize novel quantum phases. Some of these structures are predicted theoretically to host topological properties and are recognized as organic topological materials.^3–5^ In particular, MOFs often exhibit topological Dirac and flat bands, and these are commonly referred to as Dirac and flat-band (FB) materials.^6,7^ These characteristics enable systematic control of their symmetries and, consequently, their topological properties, including edge states, Hall conductivities, and related phenomena.^8–10^ Besides lattice symmetry, the symmetries of the molecular orbitals of the individual core and linker components of the MOF, are also anticipated to play a crucial role in determining the nature of the electronic bands near the Fermi level. Benefiting from the diversity of cores and linkers available for MOF formation, molecular orbital symmetry, introduces an additional DOF to tailor the characteristics of the MOF bands.^11^

Displacing ligands through alterations in the symmetry of molecular orbitals can break certain symmetries, including mirror (σ) and space inversion symmetries (SIS). Broken SIS is essential for valleytronics, as the Berry curvature (BC) vanishes in centrosymmetric materials,^12^ whereas in materials with broken SIS, a nonzero BC gives rise to the valley Hall effect (VHE). Similar to ligand displacing, another extrinsic SIS breaking can be obtained, for instance, by imposing electric field, substrate, or adsorbing atoms.^13–20^ For example, the observation of a band gap opening in epitaxial graphene^14^ is attributed to the breaking of SIS induced by the substrate potential.^15^ Moreover, mirror symmetry breaking can arise, for instance, from structural distortion, strain, or an external magnetic field.^21^

Interestingly, in addition to extrinsic effects, there are materials with intrinsic SIS breaking. Monolayers of transition-metal dichalcogenides (TMDs) are examples of two-dimensional (2D) materials with intrinsic broken SIS.^22,23^ The lack of SIS, in the presence of time-reversal symmetry (TRS) results in opposite spin orientations at the ***K*** and ***K*** ^′^ valleys, thereby locking the spin and valley DOFs. Optical control of the valley DOF can be achieved through exciton resonances that exploit valley-contrasting optical selection rules, such as excitation with σ^–^ or σ^+^ circularly polarized light.^24^

Janus variants of TMDs, provide another example of 2D materials with broken SIS, exhibiting distinct physical and chemical properties on their two faces, which is due to mirror symmetry breaking, making these materials particularly interesting.^25,26^ The experimental synthesis of Janus MoSSe via Se-to-S substitution in MoSe_2_ spurred the prediction of a broader class of 2D materials exhibiting broken SIS.^27–29^ In these 2D materials, the broken SIS creates an intrinsic electric field, which induces spin–valley splitting and Rashba-like spin splitting when coupled with spin–orbit coupling (SOC).^30–32^

At this point, it should be emphasized that the effects of spin–orbit interaction are particularly noticeable in materials without SIS, because they present spin splitting of the bands and also exhibit the nonzero anomalous velocity. It is also worth mentioning that along with SOC, exchange interactions constitute the two dominant spin-dependent interactions in solids. The exchange splitting occurs only in magnetic materials, whereas SOC is ubiquitous. The interesting aspect about SOC lifting of spin degeneracy is that it appears without magnetization. This is because appearance of spin splitting is expected for electrons moving through an effective magnetic field which is induced by an electric field perpendicular to the direction of motion. The asymmetrical potential gradient in the vicinity of atomic sites alongside spin–orbit interaction leads to spin splitting (Zeeman-like effect). Two alternative mechanisms exist for this spin–orbit spin splitting in 2D materials, related to different sources of the electric field asymmetry which are the host crystal field^33^ and the interface or surface electric field;^34^ leading to the so-called Dresselhaus and Rashba effects, respectively.

Spin and valley are here treated as additional DOFs, distinct from electronic charge, enabling exciting opportunities for semiconductor technologies. This highlights the substantial emergence of quantum phenomena, including the VHE and valley-dependent orbital magnetic moments. Recent experimental measurements have confirmed previous theoretical predictions of the VHE, and valley-dependent optical selection rules.^22^ Based on the foregoing discussion, we focus on the impact of tuning lattice, orbital, and valley DOFs via ligand substitution. With this goal in mind, and to investigate how the interplay among these DOFs influences the electronic and topological properties, we consider the Ni_3_C_12_X_12_ (X = O, S and Se) 2D MOFs and systematically substitute iso-valent ligands X in the unit cell with Y (Y = O, S and Se), and construct structures with varying compositions and configurations. For comparison, we include the Ni_3_C_12_S_12_ structure which was studied in a previous work,^35^ where we examined the effect of various DOFs on the electronic and topological characteristics. The paper is organized as follows: In Section 3.1, we present a systematic study of the electronic properties of both pristine structures and those obtained via rigid ligand substitution. Section 3.2 is devoted to the effects of this rigid ligand substitution on the topological properties of *cis*-like configurations. In Sections 3.3 and 3.4, we investigate the topological properties of *trans*-like and nonpristine homogeneous configurations, as other structures derived from rigid ligand substitution. Finally, Section 4 summarizes the main results.

## 2 Methods and Computational Details

First-principles calculations were performed using the Quantum ESPRESSO package within the framework of density functional theory (DFT).^36^ The exchange-correlation interactions were treated using the Perdew–Burke–Ernzerhof (PBE) formulation of the generalized gradient approximation (GGA).^37^ A (3 × 3 × 1) **Γ**-centered ***k***-point mesh and a plane-wave basis set with a kinetic energy cutoff of 80 Ry were employed to ensure convergence of the calculations including SOC. All calculations were performed with SOC unless otherwise specified. To further ensure that the present results are physically meaningful, we also calculated the band structures using the PBE+U functional by considering several U values, including U = 0, 3, and 5 eV,^38^ and the corresponding results are presented in Figure S1. As shown, the target Kagome bands remain largely unaffected by the inclusion of the U parameter, indicating that the choice of U has only a minor influence on the overall characteristics of the target Kagome bands. These results are in good agreement with the conclusions reached by previous first-principles studies.^39,40^

The monolayer Ni_3_C_12_X_12_ was modeled using a slab geometry with a vacuum spacing of 12 Å to eliminate interlayer interactions. The unit cell comprises 27 atoms, including 3 Ni, 12 C, and 12 X atoms. The projector augmented-wave (PAW) method was used to describe the interaction between core electrons and the valence electron density,^41^ and atomic positions for pristine structures were relaxed until the forces on each atom were below 0.002 Ry/Bohr.

Berry curvature and spin Hall conductivity (SHC) were evaluated using the WANNIER90 code,^42^ with a 3 × 3 × 1 ***k***-point grid for Wannier interpolation. The SHC was computed using the post processing module (BERRY) of the WANNIER90, as developed by Qiao et al.^43^ by integrating the Berry-like curvature over the Brillouin zone (BZ).^44^ For accurate evaluation of the SHC, a dense 500 × 500 × 500 ***k***-mesh was used and to deal with the rapid variation of the SHC, an adaptive refinement ***k***-mesh of 4 × 4 × 4 was employed. The method of adaptive ***k***-mesh refinement can be effective for an efficient convergence of SHC calculation.^43^

To analyze the edge states and Z_2_ invariants we employed the WANNIER90 package to construct Wannier functions based on nickel *d*_*xz*_, *d*_*yz*_ orbitals, carbon *p*_*z*_ orbitals and X, Y ligands *p*_*z*_ orbitals. These were then used in conjunction with surface Green’s function techniques as provided by the WANNIERTOOLS software.^45^

Using the computational tools and theoretical framework described in the following section, we systematically investigated key topological properties, including the SHC, spin Berry curvature (SBC) and BC. These analyses provide deeper insight into the relationship between the electronic structure and topological features in topological quantum materials.

The Kubo formula for the transverse conductivity in the direct current (dc) limit is expressed in terms of the SBC,^46^ as expressed in eq 1

$$\sigma_{\alpha \beta }^{\gamma }=-\frac{e^{2}}{\hbar }\int_{BZ}\frac{dk}{{\left(2\pi \right)}^{2}}\Omega_{\alpha \beta }^{\gamma }\left(k\right)$$ 1

the total ***k***-resolved SBC, Ω_*αβ*_^γ^(***k***), is defined in eq 2

$$\Omega_{\alpha \beta }^{\gamma }\left(k\right)=\underset{n}{\sum }f\left(E\right)\Omega_{n,\alpha \beta }^{\gamma }\left(k\right)$$ 2

where the sum is over *n* bands, *f*(*E*) = 1/[*e* ^(*E*–μ)/(*k*_*B*_T)^ + 1] is the Fermi–Dirac distribution function with μ, *k*_*B*_, and T, the chemical potential, Boltzmann constant, and absolute temperature, respectively. The Fermi–Dirac factor restricts the sum to occupied bands. The band-projected spin Berry curvature-like term is defined as in eq 3 ^43^

Ωn,αβγ(k)=−2ℏ2Im⁡∑m≠n⟨ψnk|J^αγ|ψmk⟩⟨ψmk|ν̂β|ψnk⟩(ϵnk−ϵmk)2 3

where, ϵ_*n* ***k***_ and ϵ_*m* ***k***_ are the eigenvalues corresponding to the Bloch eigenstates |ψ_*n* ***k***_⟩ and |ψ_*m* ***k***_⟩. The spin-velocity operator is defined as J^αγ={σγ,ν̂α}/2 where *ν̂*_*i*_ = ℏ^–1^ *∂Ĥ*/*∂* ***k*** *_i_* with i = α, β is the velocity operator (*Ĥ* is the Hamiltonian), and *σ*_*γ*_ with γ = *x, y, z* are the Pauli matrices. Taking upper index (γ = *c*) as charge DOF and *σ*_*c*_ as a (2 × 2) identity matrix, we find J^αc=ν̂α and can define ordinary Berry curvatures (BC) in the same manner as SBC. In 2D, the BC possesses only one component and is defined as eq 4 ^47^

Ωn,xyc(k)=−2Im⁡∑m≠n⟨ψnk|ν̂x|ψmk⟩⟨ψmk|ν̂y|ψnk⟩(ϵnk−ϵmk)2 4

## 3 Results and Discussion

As a result of structural relaxation, the studied pristine structures Ni_3_C_12_X_12_ with (X = O, S and Se) exhibit a planar configuration and 6-fold symmetry, like experimentally synthesized structure Ni_3_C_12_S_12_.^48^ Their electronic ground state corresponds to a nonmagnetic insulator,^38^ and first-principles simulations demonstrated their thermal^49^ and dynamical stability.^39,50^ The optimized lattice constants of the Ni_3_C_12_X_12_ monolayers with (X = O, S and Se), are *a* = *b* = 12.99 Å, 14.64 Å and 15.35 Å, respectively, which agree well with both recent experimental observations^48^ and earlier DFT studies.^49^ The metal sites in these M_3_L_2_-type MOFs (M = Ni and L = C_6_X_6_) have a local structure of four neighboring atoms in a planar configuration regardless of the type of ligand molecules. For example, any Ni atom in Ni_3_C_12_X_12_ (see Figure 1a) is surrounded by four X atoms.^38^ Accordingly, Ni_3_C_12_X_12_ is classified as a hexagonal crystal with SIS. The symmetry of the crystal corresponds to the *P6/mmm* (No. 191) space group, whose point group is *D*_*6h*_. Its crystal structure involves a 3-fold rotational symmetry (*C*_*3z*_), the vertical mirror symmetries, the horizontal mirror symmetry, and the SIS. Figure 1a illustrates the unit cell structure of a M_3_L_2_-type Kagome lattice. In this configuration, the metal atoms create the Kagome lattice, while the organic ligands (C_6_X_6_) are positioned within the metal triangles.

**Figure 1:**
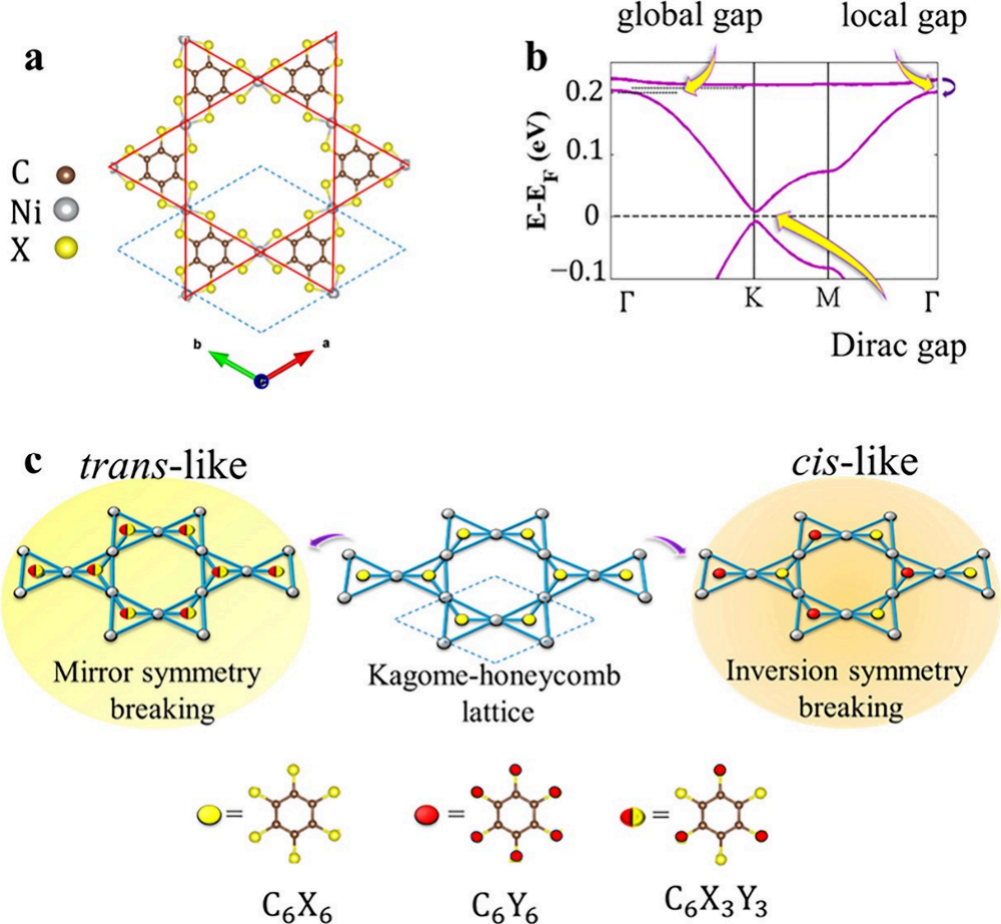
(a) Crystal structure of the Ni_3_C_12_X_12_ (X = O, S, Se) featuring Kagome-honeycomb lattice outlined by red solid lines. The blue dashed lines indicate the unit cell defined by the lattice vectors **a** and **b**. C atoms are shown in brown, Ni in gray, and X in yellow. (b) Showing three SOC gaps containing global, local and Dirac gaps in a typical Kagome lattice. (c) Central panel displays a typical Kagome-honeycomb composite lattice and the blue dashed lines indicate the unit cell. The right panel illustrates a *cis*-like configuration with broken SIS and the left panel illustrates a *trans*-like configuration with broken some mirror symmetries. Red circles represent C_6_Y_6_, yellow circles represent C_6_X_6_, and red-yellow circles are C_6_X_3_Y_3_ ligands, which forming the honeycomb sublattice. The gray circles which indicate Ni atoms, comprising the Kagome sublattice.

Through rigid systematic substitution of ligand X with Y (where X and Y = O, S or Se) twenty-one Ni_3_C_12_X_6_Y_6_ structures were generated, which can be experimentally realized using approaches similar to those employed for comparable structures like MoSSe, a 2D Janus monolayer.^51,52^ By changing the orbital DOF, we refer to the incorporation of selenium atoms into certain structures, since selenium possesses fully occupied *d*-orbitals, unlike sulfur and oxygen. These structures can be classified into three groups. Two groups of these structures exhibit *cis*- and *trans*-like configurations, with 50% ligand replacement and are denoted as I, II and III­(NiX_2_Y_2_)_c, t_ where X represents the host and Y indicating the guest ligands with Y ≠ X. The I, II, III symbols are used to indicate unit cell areas of 146.07 Å^2^, 185.64 Å^2^, and 204.04 Å^2^, corresponding to Ni_3_C_12_O_12_, Ni_3_C_12_S_12_ and Ni_3_C_12_Se_12_, respectively. The subscripts ‘c’ and ‘t’ denote *cis*- and *trans*-like configurations, respectively. The corresponding *cis*- and *trans*-like structures include I­(NiO_2_S_2_)_t_, I­(NiO_2_S_2_)_c_, I­(NiO_2_Se_2_)_t_, I­(NiO_2_Se_2_)_c_, II­(NiS_2_O_2_)_t_, II­(NiS_2_O_2_)_c_, II­(NiS_2_Se_2_)_t_, II­(NiS_2_Se_2_)_c_, III­(NiSe_2_O_2_)_t_, III­(NiSe_2_O_2_)_c_, III­(NiSe_2_S_2_)_t_, III­(NiSe_2_S_2_)_c_. The third group of I, II and III­(NiX_2_Y_2_) systems have (X = Y = O, S and Se) where all ligands are replaced; these structures are therefore referred to as homogeneous and are designated as I­(NiO_4_), I­(NiS_4_), I­(NiSe_4_), II­(NiO_4_), II­(NiS_4_), II­(NiSe_4_), III­(NiO_4_), III­(NiS_4_), III­(NiSe_4_). Some of these homogeneous structures—namely I­(NiO_4_), II­(NiS_4_), and III­(NiSe_4_) previously introduced as Ni_3_C_12_O_12_, Ni_3_C_12_S_12_, and Ni_3_C_12_Se_12_— are referred here as pristine structures. This terminology is adopted to maintain consistency with the naming of other structures. The naming convention is based on the spatial arrangement of four atoms surrounding the nickel center. For the 18 nonpristine structures, we performed rigid ligand substitutions (replacing O with S/Se and vice versa) within a fixed unit cell, without relaxing either the atomic positions or the lattice parameters. This approach intentionally preserves the maximum strain induced by ligand size mismatch, thereby maintaining the system under artificial strain. The three reference unit cell areas (I, II, and III) were selected as controlled templates to systematically vary the global strain level, while applying local substitutions. To further investigate the effect of this artificial constraint, we consider the results of a full structural relaxation. These showed that the monolayer thickness remained essentially unchanged, and no buckling of the structure was observed.

As a result of these substitutions, certain symmetries are either broken or preserved, and various structural tensions are introduced. For instance, in *cis*-like configurations SIS is broken, whereas in *trans*-like configurations it is preserved—though some mirror symmetries are disrupted instead.

A typical 2D MOF with a Kagome-honeycomb lattice can be modeled as a composite structure consisting of a Kagome sublattice of heavy transition metal ions (gray circles in Figure 1c) and a honeycomb sublattice of C_6_X_6_ groups (X = O, S or Se; yellow circles in Figure 1c). This figure schematically illustrates the construction of both *cis*- and *trans*-like configurations. Replacing one of the two C_6_X_6_ groups in the unit cell with a C_6_Y_6_ group (red circles, Figure 1c), where Y differs from X, results in a *cis*-like configuration with broken SIS. In this arrangement, one side of the unit cell contains a C_6_X_6_ group, while the opposite side contains a C_6_Y_6_ group. Conversely, the *trans*-like configuration (left panel, Figure 1c), retains only a horizontal mirror plane (the plane of the crystal structure) while breaking vertical mirror symmetries. In this case, the C_6_X_3_Y_3_ groups occupy both sides of the unit cell, with X and Y ligands arranged in an alternating fashion. The structures corresponding to the red, yellow, and bicolored circles are depicted at the bottom of the Figure 1c, and the further structural details provided in Figure S2 of the Supporting Information (SI).

As previously mentioned, replacement of iso-valent ligands induces a form of structural tension, referred here as “ligand-decorated induced local tension”.^53^ For instance, in *cis*- and *trans*-like configurations, beyond symmetry breaking, rigid ligand substitution also induces structural tension within the newly formed structures. In contrast, nonpristine homogeneous structures exhibit only tension, with no observable symmetry reduction. A measure of the magnitude of this tension is reported in Figure 2 for the twenty-one structures. The measure is based on the “bond length deviation” (BLD) concept defined as in eq 5, where *L*_*pr*_ and *L* represent the pristine and final bond lengths, respectively; the first corresponding to the value without ligand replacement, *N* is the number of altered bonds; and L*_c_* denotes the lattice constant value.

$$\mathrm{BLD}=\frac{\overset{N}{\underset{i=1}{\sum }}\left(L-L_{pr}\right)}{N\times L_{c}}\times 100$$ 5

**Figure 2:**
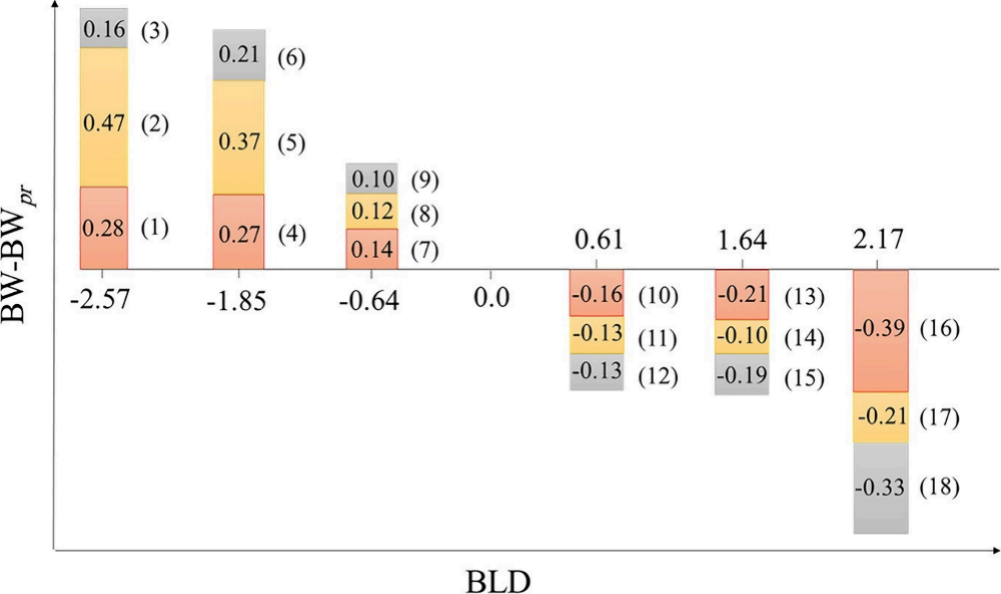
Variation in Kagome bands width (BW) at the **Γ** point relative to the pristine value (BW*_pr_*) for all studied structures as a function of bond length deviation (BLD). The pristine bandwidth and the zero BLD, correspond to that of the pristine structures I­(NiO_4_), II­(NiS_4_), and III­(NiSe_4_). The numbers correspond to (1) I­(NiSe_4_), (2) I­(NiO_2_Se_2_)_c_, (3) I­(NiO_2_Se_2_)_t_, (4) I­(NiS_4_), (5) I­(NiO_2_S_2_)_c_, (6) I­(NiO_2_S_2_)_t_, (7) II­(NiSe_4_), (8) II­(NiS_2_Se_2_)_c_, (9) II­(NiS_2_Se_2_)_t_, (10) III­(NiS_4_), (11) III­(NiSe_2_S_2_)_c_, (12) III­(NiSe_2_S_2_)_t_, (13) II­(NiO_4_), (14) II­(NiS_2_O_2_)_c_, (15) II­(NiS_2_O_2_)_t_, (16) III­(NiO_4_), (17) III­(NiSe_2_O_2_)_c_, and (18) III­(NiSe_2_O_2_)_t_.

The values used to estimate the dimensionless BLD for these twenty-one structures were based on the bond lengths of Ni – X, C – X, C – C and the lattice constant of the Ni-based MOFs. It is to be noted that, although the *cis*- and *trans*-like configurations are subject to equal tension, they differ in symmetry. As a result, *cis*- and *trans*-like structures display distinct electronic and topological properties.

In *trans*-like configurations, the presence of different ligands induces mirror symmetry breaking and reduces the crystal symmetry to space group *P6/m* (#175) and the point group to *C*_*6h*_, a subgroup of *D*_*6h*_. In contrast, *cis*-like configurations break SIS, leading to a reduction of the crystal symmetry to space group *P* 6̅ *2/m* (#189) and the point group to *D*_*3h*_, resulting in noncentrosymmetric structures. Figure S3 illustrates the SIS breaking in a *cis-*like structure.

To assess the stability of the pristine monolayer structures, the cohesive energy (*E*_C_) was calculated as in eq 6

$$E_{C}=\frac{E_{{\mathrm{Ni}}_{3}C_{12}X_{12}}-\left(N_{\mathrm{Ni}}E_{\mathrm{Ni}}+N_{C}E_{C}+N_{X}E_{X}\right)}{N}$$ 6

where E_Ni_3_C_12_X_12__ is the total energy of the desired pristine monolayers; E_Ni_, E_C_ and E_X_ are the total energies of isolated Ni, C and X atoms, respectively, computed including spin polarization and with the atoms in an asymmetric box to ensure the appropriate occupation. Here, N stands for the total number of atoms in the monolayer structures, N_Ni_, N_C_ and N_X_ represent the number of Ni, C and X (O, S, Se). The cohesive energies of these investigated structures are summarized in Table S1. Note that the cohesive energy values are generally comparable to those of the stable graphene structure.

### 3.1 Electronic Properties of Configurations with Different Geometrical Symmetries

In this section we consider the band structure of each of the twenty-one configurations obtained by substituting iso-valent ligands and excluding SOC (see Figure S4). This figure reveals that in the configurations composed exclusively of oxygen ligands— I­(NiO_4_), II­(NiO_4_) and III­(NiO_4_)— there are four (purple) distinct Kagome bands, whereas in all other cases only three Kagome bands appear. Conversely, in other structures containing oxygen—such as I­(NiO_2_S_2_)_c_, I­(NiO_2_S_2_)_t_, I­(NiO_2_Se_2_)_c_, I­(NiO_2_Se_2_)_t_, II­(NiS_2_O_2_)_c_, II­(NiS_2_O_2_)_t_, III­(NiSe_2_O_2_)_c_ and III­(NiSe_2_O_2_)_t_—the reduction in symmetry leads to the lifting of degeneracy between the lower FB and the Dirac bands, resulting in their separation and the emergence of three Kagome bands.

The Kagome bands width (BW) at the **Γ** point for the twenty-one structures varies depending on the sign of the induced tension, either increasing or decreasing relative to the Kagome BW of their pristine counterparts (BW*_pr_*). The corresponding Kagome BW values are provided in Table S2. Specifically, in structures subjected to positive tension (tensile), the Kagome BWs are reduced compared to those of the pristine configurations (BW-BW*_pr_* < 0), as clearly shown in Figure 2. This is the case of II­(NiO_4_), II­(NiS_2_O_2_)_c_, II­(NiS_2_O_2_)_t_, III­(NiO_4_), III­(NiSe_2_O_2_)_c_ III­(NiSe_2_O_2_)_t_, III­(NiS_4_), III­(NiSe_2_S_2_)_c_ and III­(NiSe_2_S_2_)_t_ structures, additional details are provided in Figure S4. Among the structures that exhibit a decrease in Kagome BW, only II­(NiO_4_), III­(NiO_4_) and III­(NiS_4_) display this reduction as a direct consequence of pure tension, independent of symmetry breaking effects.

In contrast, I­(NiS_4_), I­(NiO_2_S_2_)_c_, I­(NiO_2_S_2_)_t_, I­(NiSe_4_), I­(NiO_2_Se_2_)_c_, I­(NiO_2_Se_2_)_t_, II­(NiSe_4_), II­(NiS_2_Se_2_)_c_ and II­(NiS_2_Se_2_)_t_ structures subjected to negative tension (compressive), exhibit an increase in Kagome BW relative to their pristine counterparts (BW-BW*_pr_* > 0), as also shown in Figure 2. Among these structures, only the I­(NiS_4_), I­(NiSe_4_) and II­(NiSe_4_) exhibit a BW increase attributable solely to the induced local tension. Notably, in homogeneous structures, any observed variations in electronic behavior can be attributed solely to the induced local tension. In contrast, for structures with partial ligand substitution (*cis*- and *trans*-like configurations), both the induced tension and the reduction in structural symmetry contribute to the observed modifications in electronic behavior. Note that Kagome BW values are related to the hopping parameters,^11^ and can provide information about their magnitude.

As illustrated in Figure 3, the SIS breaking in *cis*-like configurations leads to the emergence of a gapped Dirac cone, indicative of massive Dirac Fermions (see also Figure S4). The broken SIS induces a valley Hall gap in these configurations, consistent with phenomena reported in previous works.^54,55^

**Figure 3:**
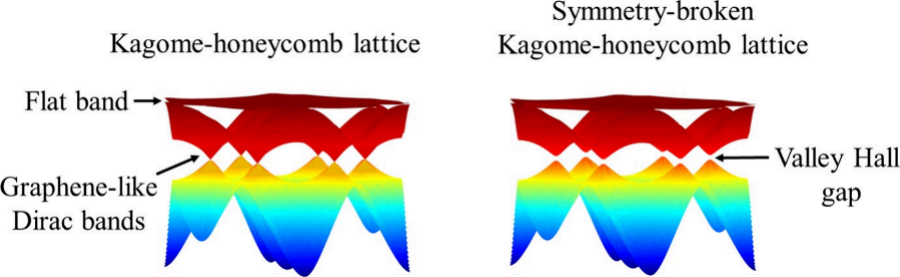
3D band structures of the Kagome-honeycomb lattice and its symmetry-broken counterpart. Illustrates the Dirac cone and nearly FB in the Kagome-honeycomb lattice (left) and gapped Dirac cone in the symmetry-broken Kagome-honeycomb lattice (right), leading to the emergence of a valley Hall gap.

The projected density of states (PDOS) diagram for the twenty-one distinct structures shows that the states above the Fermi level correspond to the Kagome bands, which originate from the *p*_*z*_ orbitals of the carbon atoms, two *d*_*xz*_ and *d*_*yz*_ orbitals of the nickel atoms, and the *p*_*z*_ orbitals of the X and Y ligands. In systems containing two heterogeneous ligands (X and Y) arranged in *cis*- and *trans*-like configurations, the atoms (X or Y) with lower electronegativity contributes more prominently to the density of accessible states within the Kagome bands (see Figure S5).

Among the twenty-one structures, nine exhibit metallic behavior, including I­(NiO_4_), I­(NiO_2_S_2_)_c_, I­(NiO_2_Se_2_)_c_, II­(NiO_4_), II­(NiS_2_O_2_)_c_, II­(NiS_2_O_2_)_t_, III­(NiO_4_), III­(NiS_4_) and III­(NiSe_2_O_2_)_c_, while the remaining 12 structures can be classified as semiconductors. The PDOS plots reveal that, in structures incorporating oxygen ligands, the electronic states are more localized, with notably weaker charge distribution and interatomic interactions compared to those observed in structures containing sulfur and selenium ligands.

Bader charge analyses,^56,57^ carried out with the tools developed by Henkelman et *al.*,^58^ confirm the distinct behavior of structures containing oxygen ligands. Analysis of Bader charges for O, S and Se across the twenty-one structures reveals that O atoms gain electron density and, in all cases, exhibit a negative charge (see Figure S6). This behavior, attributed to the high electronegativity of O, reduces its orbital overlap with neighboring atoms and consequently diminishes its participation in interactions.

In the band structures of the twenty-one configurations calculated including SOC, one observes that in most cases, SOC induces three distinct nontrivial band gaps, as illustrated in Figure 1b: a Dirac band gap at the ***K*** point, a global band gap between the minimum of nearly FB and the maximum of the upper Dirac band, and a local band gap at the **Γ** point (see Figure S7 and Table S3). It is evident that the application of tensile tension in structures II­(NiO_4_) and III­(NiO_4_) not only results in a narrowing of the Kagome BW relative to the pristine structure I­(NiO_4_), but also induces an additional nontrivial band gap between the lower nearly FB and the lower Dirac band. The magnitude of this new gap, absent in the pristine structure I­(NiO_4_), is 28.0 and 27.8 meV for II­(NiO_4_) and III­(NiO_4_) structures, respectively (see Figure S7). Another noteworthy observation is that the configurations including I­(NiO_4_), I­(NiSe_4_), I­(NiO_2_S_2_)_c_, I­(NiO_2_S_2_)_t_, I­(NiO_2_Se_2_)_c_, I­(NiO_2_Se_2_)_t_, II­(NiO_4_), II­(NiS_2_O_2_)_c_, II­(NiS_2_O_2_)_t_, III­(NiO_4_), III­(NiS_4_), III­(NiSe_2_O_2_)_c_ and III­(NiSe_2_O_2_)_t_ do not exhibit global band gaps. Notably, most of these configurations contain O atoms. The analyses of the band structures also reveals that all structures require electron doping to shift the Fermi level into the nontrivial Dirac band gaps (see Figure S8 as an example, for the band structure analysis of II­(NiS_4_) at different electron doping concentrations). In particular, the electron doping concentrations for the structures labeled with indices (I, II, and III) are (1.37 × 10^14^, 1.07 × 10^14^ and 0.98 × 10^14^) cm^–2^ corresponding to the addition of two electrons per unit cell in each case. For details on nontrivial band gaps in electron doped structures, see Table S4. We used superscript “-2” to denote these electron-doped structures. The cohesive energies of the pristine structures under the specified electron doping concentrations are summarized in Table S5.

Another notable effect observed in the *cis*-like configurations, which lack SIS is that incorporating SOC lifts the degeneracy of the Kagome bands at specific points in the BZ, particularly at the (***K*** and ***K*** ′). This lifting occurs because these points are not protected by TRS. In contrast, at high-symmetry points such as **Γ** and **M**, where TRS is preserved, this degeneracy remains intact. These symmetry-protected points are referred to as time-reversal invariant momenta (TRIM). The preserved degeneracy at these points is known as Kramer’s degeneracy and is usually expressed as [E^↑^(***k***) = E^↓^(***k***)]. Kramer’s degeneracy is maintained in systems that possess both TRS, [E^↑^(***k***) = E^↓^(−***k***)], and SIS, [E^↑^(***k***) = E^↑^(−***k***)].

Lifting of spin degeneracy in *cis*-like configurations by accounting for SOC, leads to Zeeman-like spin splitting of the energy bands at the ***K*** and ***K*** ′ points. Although upon including SOC, the spin-up and spin-down bands are no longer separable, the out-of-plane spin component (ŝ*_*z*_*) remains approximately a good quantum number near the ***K*** and ***K*** ′ valleys. Accordingly, the ŝ*_*z*_* projected band structure of *cis*-like configurations is derived by projecting the spin operator ŝ*_*z*_* in the ψ_*n* ***k***_ manifold—i.e. ⟨ψ_*n* ***k***_ | ŝ*_*z*_* | ψ_*n* ***k***_⟩— obtained via Wannier interpolation.^59^ In other words, the band structure is mapped by ŝ*_*z*_* for these *cis*-like structures (see Figure S9). Generally, in the presence of SOC, SIS breaking lifts the spin degeneracy at the ***K*** and ***K*** ′ valleys. However, due to TRS, the spin splitting at these two valleys exhibits opposite sign. As a result, spin DOF is locked with the valley index. As an illustrative example, Figure 4a presents the relativistic band structure of II­(NiO_2_S_2_)_c_^–2^ structure and a schematic view of the spin splitting at the ***K*** and ***K*** ′ valleys is shown in the AI generated^60^ Figure 4b, which clearly illustrates the reversed spin splitting in opposite valleys across three bands. As a result, spin moments can act as identifiers of valley carriers, forming the basis for coupled spin–valley physics.

**Figure 4:**
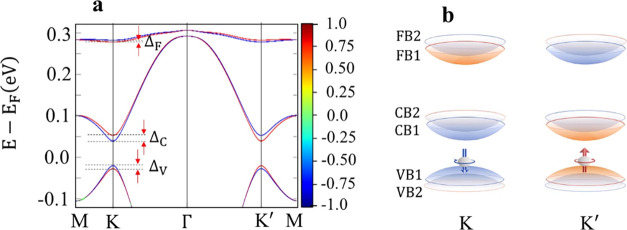
(a) Relativistic band structure of the II­(NiO_2_S_2_)_c_^–2^ with the projection of the spin operator ŝ_*z*_. The red and blue colors represent spin-up and spin-down states, respectively. The color scale indicates the expectation value ⟨ŝ_*z*_⟩ of the spin operator ŝ_*z*_ in units of *ℏ*/2. Spin splitting at conduction, valence and nearly flat band are shown by Δ_C_, Δ_V_, and Δ_F_ (b) Schematic illustration of spin splitting with opposite signs at the ***K*** and ***K*** ′ valleys. VB1, VB2 are the highest occupied band and the second highest occupied band. CB1 and CB2 are the lowest unoccupied band and the second lowest unoccupied band, respectively. FB1 and FB2 are the lowest unoccupied nearly flat band and the second lowest unoccupied nearly flat band, respectively.

In the context above, a direct band gap is observed at two inequivalent valleys, ***K*** and ***K*** ′. Notably, in all *cis*-like configurations, spin splitting occurs both at the valence band (VB) and conduction band (CB). In contrast, in materials such as monolayer MoS_2_, which also exhibit broken SIS,^61–63^ spin splitting is observed only at the VB. The magnitude of spin splitting is generally attributed to the strength of SOC, with larger splitting enhancing a material’s potential for spintronic applications.^64^ Table 1 summarizes the spin splitting values at the ***K*** point for six *cis*-like configurations. Relative to the other structures, spin splitting with opposite sign in the ***K*** and ***K*** ′ valleys, is particularly pronounced in two configurations, II­(NiS_2_O_2_)_c_^–2^ and III­(NiSe_2_O_2_)_c_^–2^, see Figure S9. Notably, these two configurations also display the largest SOC band gaps and the highest anionic inner potential differences, which will be further discussed in the next section. The valley spin splitting for the valence, conduction and nearly flat bands are expressed as Δ_V_^***K***/***K*** ^′^^, Δ_C_^***K***/***K*** ^′^^ and Δ_F_^***K***/***K*** ^′^^, respectively and are defined in eq 7

$$\Delta_{V}=E_{K}^{\mathrm{VB}1}-E_{K}^{\mathrm{VB}2},\Delta_{C}=E_{K}^{\mathrm{CB}2}-E_{K}^{\mathrm{CB}1}\;\mathrm{and}\;\Delta_{F}=E_{K}^{\mathrm{FB}2}-E_{K}^{\mathrm{FB}1}$$ 7

where, E_***K***_^VB1^ and E_***K***_^VB2^ are the highest occupied band energy and the second highest occupied band energy at the ***K*** valley. Note also that, due to TRS, Δ_V_^***K***^ (Δ_C_^***K***^, Δ_F_^***K***^) is equal to Δ_V_^***K*** ^′^^(Δ_C_^***K*** ^′^^, Δ_F_^***K*** ^′^^).

**Table 1:** Spin Splitting (in meV) at Conduction, Valence, and Nearly Flat Band (Δ_C_, Δ_V_, and Δ_F_, Respectively) at the *K* Point for Six *Cis*-Like Configurations in the Presence of SOC

structure	I(NiO_2_S_2_)_c_^–2^	I(NiO_2_Se_2_)_c_^–2^	II(NiS_2_O_2_)_c_^–2^
Δ_C_	14.0	12.0	20.0
Δ_V_	6.0	6.4	10.0
Δ_F_	4.3	3.0	10.0

structure	II(NiS_2_Se_2_)_c_^–2^	III(NiSe_2_O_2_)_c_^–2^	III(NiSe_2_S_2_)_c_^–2^
Δ_C_	10.0	20.0	20.0
Δ_V_	10.0	20.0	10.0
Δ_F_	0.0	10.0	0.0

The largest spin splitting is observed in the III­(NiSe_2_O_2_)_c_^–2^ configuration, with a maximum spin splitting of 20 meV at both the VB and the CB. This significant splitting can be attributed to the substantial anionic inner potential difference in this structure. Similarly, the II­(NiS_2_O_2_)_c_^–2^ and III­(NiSe_2_S_2_)_c_^–2^ structures also display a spin splitting of up to 20 meV at their CB. Another notable observation is the presence of spin splitting in the nearly FB, in addition to that in the VB and the CB. This FB spin splitting is evident in four of *cis*-like configurations, excluding the two II­(NiS_2_Se_2_)_c_^–2^ and III­(NiSe_2_S_2_)_c_^–2^ structures. The absence of FB spin splitting can be attributed to the relatively smaller anionic inner potential difference in these structures compared to the other four structures.

To elucidate the distinct behavior observed in *cis*-like configurations, the plane-averaged (planar) electrostatic potential was computed for these structures. The resulting nonuniform potential^20^ arises from the asymmetry of the *cis*-like configurations, which induces an asymmetric distribution of charge density around the Ni atoms. This asymmetric potential breaks SIS, thereby facilitating the opening of a valley Hall gap. Figure 5 illustrates the variation in the anionic inner potential across six *cis*-like configurations.

**Figure 5:**
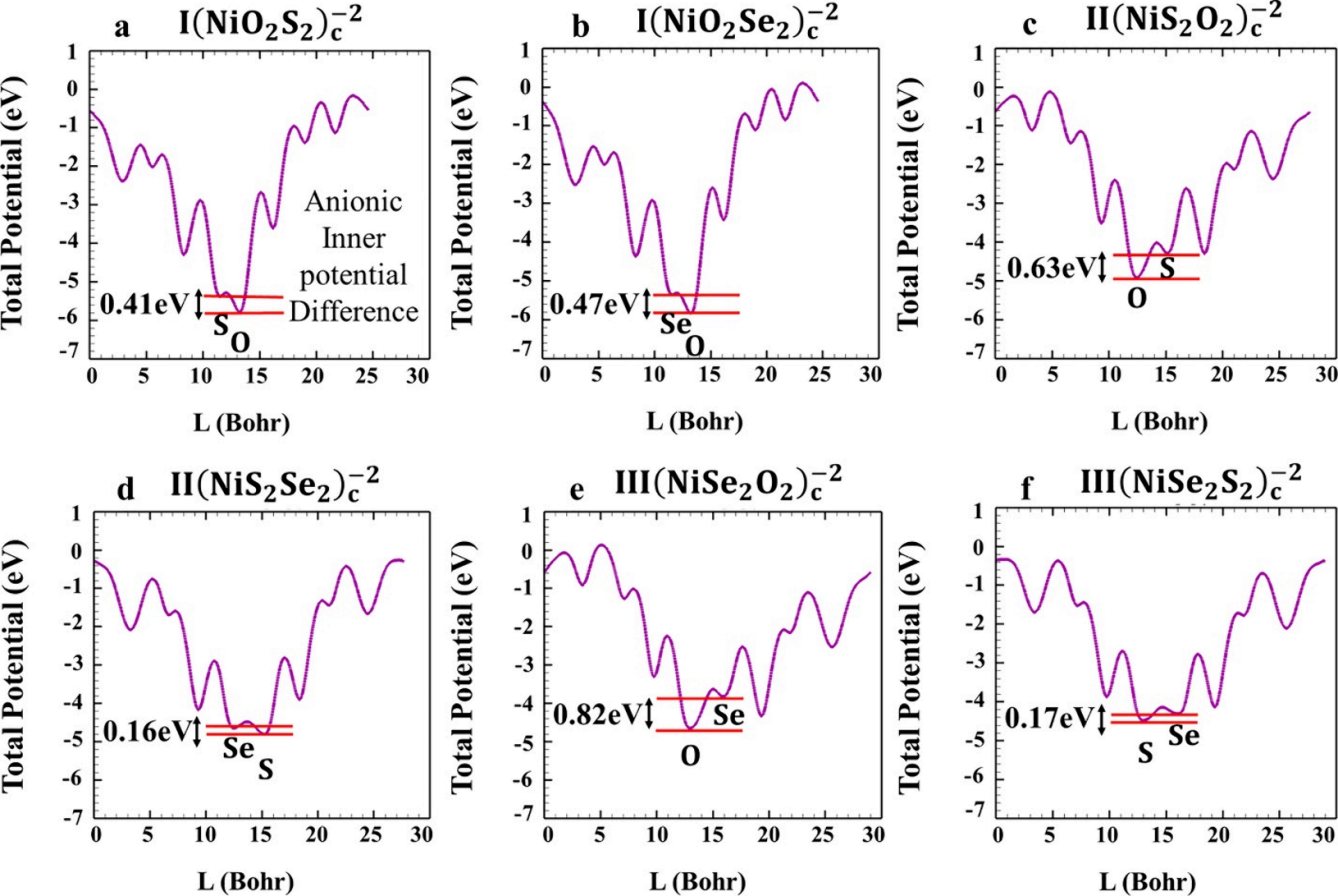
Planar electrostatic potential profiles along the *x*-axis for structures exhibiting *cis*-like configurations for (a) I­(NiO_2_S_2_)_c_^–2^, (b) I­(NiO_2_Se_2_)_c_^–2^, (c) II­(NiS_2_O_2_)_c_^–2^, (d) II­(NiS_2_Se_2_)_c_^–2^, (e) III­(NiSe_2_O_2_)_c_^–2^, and (f) III­(NiSe_2_S_2_)_c_^–2^.

Beyond their differing symmetries, and despite possessing an equal number of hosts–guest ligands, the fundamental distinction between *cis*- and *trans*-like configurations lies in the variation of their electrostatic potential or charge density distributions. The calculated average electrostatic potential along the *x*-axis in the *cis*-like configurations, exhibits a pronounced asymmetry whereas the potential remains symmetric in both *trans*-like and pristine structures (see Figure S10).

The III­(NiSe_2_O_2_)_c_^–2^ configuration exhibits the highest anionic inner potential difference of 0.82 eV, whereas II­(NiS_2_Se_2_)_c_^–2^ and III­(NiSe_2_S_2_)_c_^–2^ display the lowest values of 0.16 and 0.17 eV, respectively. Notably, the substantial anionic potential difference correlates directly with the largest SOC band gap and the broadest BC distribution at the ***K*** point, as will be discussed in subsequent sections. Figure 5 clearly shows that the electrostatic potential is minimized at the center of these configurations, particularly on the side where the ligand exhibits a lower potential and, hence, higher electronegativity. For instance, in the III­(NiSe_2_O_2_)_c_^–2^, the electrostatic potential is significantly more negative on the O side than on the Se side, expressed as (*V*_O_ < *V*_Se_), which is consistent with previous findings.^65,66^ Following the analysis of electronic properties, the topological characteristics of the *cis*-, *trans*-like and homogeneous configurations —excluding the pristine I­(NiO_4_)^−2^, II­(NiS_4_)^−2^ and III­(NiSe_4_)^−2^ structures— are systematically investigated.

### 3.2 Topological Properties of *Cis*-Like Configurations

Following well-established methodologies in the study of topological phases,^35^ the contributions of individual atoms to the formation of the Kagome (target) bands were identified and used as initial projections for the construction of Wannier functions. This approach—Wannierization based on a fixed number of target bands—is widely employed for investigating topological properties.^67–70^ Subsequently, tight-binding Hamiltonians are derived from the maximally localized Wannier functions (MLWFs), which are iteratively obtained by minimizing the spread of the localization functional, to compute specific topological properties, including BC, SHC, edge states, and topological invariants.

When a crystal possesses both SIS and TRS, the BC vanishes throughout the BZ.^71^ However, breaking SIS —such as in *cis*-like configurations— induces a finite BC with opposite signs in the inequivalent valleys (***K*** and ***K*** ′), as clearly shown in Figure 6. Under these conditions, charge carriers experience valley-contrasting BC, Ω_*xy*_^*c*^ (***k***), which plays a crucial role in valley-dependent transport phenomena.^72^

**Figure 6:**
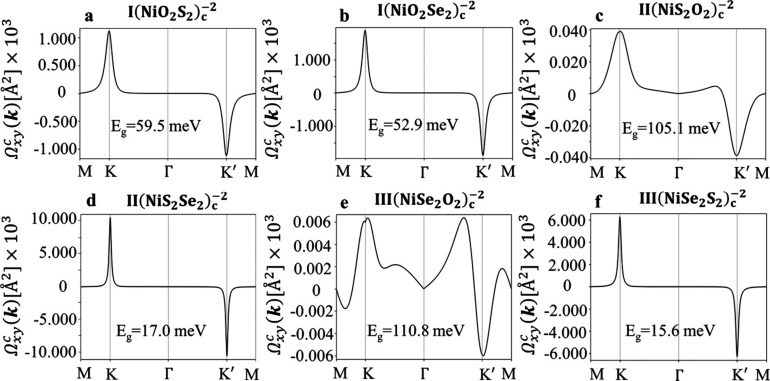
Berry curvature of the VB in *cis*-like configurations I, II, and III (NiX_2_Y_2_), along the high-symmetry lines. The results correspond to structures with two electron doping concentrations, where the Fermi level is located within the Dirac band gap for (a) I­(NiO_2_S_2_)_c_^–2^, (b) I­(NiO_2_Se_2_)_c_^–2^, (c) II­(NiS_2_O_2_)_c_^–2^, (d) II­(NiS_2_Se_2_)_c_^–2^, (e) III­(NiSe_2_O_2_)_c_^–2^, and (f) III­(NiSe_2_S_2_)_c_^–2^. E_g_ denotes the corresponding SOC-induced band gap.

To verify the nonequivalence of the valleys at the ***K*** and ***K***, we have analyzed the nonzero component of BC. In the presence of a nonzero BC, the velocity of charge carriers is expressed as ***ϑ*** *_n_* (***k***) = ***ϑ***_g_ – ***ϑ***_⊥_. In this context, ***ϑ*** *_g_* represents the group velocity, defined as $\vartheta_{g}=\frac{1}{\hbar }\nabla_{k}\varepsilon_{n}\left(k\right)$ where *ε*_*n*_(***k***) is the energy of the |ψ_*n* ***k***_⟩ state. BC introduces a transverse velocity component, ***ϑ***_⊥_, in addition to the group velocity. This transverse anomalous velocity is defined as $\vartheta_{⊥}=-\frac{e}{\hbar }E\times \Omega_{n}\left(k\right)$ , where ***E*** represents the in-plane electric field and *Ω*_*n*_ (***k***) signifies the out-of-plane BC of the |ψ_*n* ***k***_⟩ state. Consequently, carriers at the ***K*** and ***K*** ′ valleys acquire opposite anomalous velocities, driving them toward opposite boundaries. Figure 6 illustrates that the BC reaches its maximum value at the valleys ***K*** and ***K*** ′, where the Fermi level crosses the SOC band gaps, with opposite signs (due to the presence of TRS, Ω_*xy*_^*c*^ (***K***) = – Ω_*xy*_^*c*^ (***K*** ′)). However, its value decreases as moving away from these valleys, rapidly decaying and vanishing at the **Γ** and **M** points. The larger BC can amplify the transverse velocity, thereby enabling faster charge transport and reduced carrier recombination. Consequently, a larger Ω_*xy*_^*c*^ (***k***) is highly desirable, making the modulation of BC a critical aspect in the field of valleytronics.^73^ There is an inverse relationship between the band gap and the magnitude of the BC.^12,74^ Namely, the smaller the SOC gaps (E_g_ at Figure 6), the larger the resulting BC. As shown in Figure 6, the width of the BC peak (the cross-section) at the BZ corners ***K*** and ***K*** ′ is directly correlated with the band gap at the Dirac point, denoted as E_g_. The ***k***-space contrasting Ω_*xy*_^*c*^ (***k***) in structures without SIS is a key quantity for characterizing valley-contrasting phenomena, such as VHE.^12,72^

Furthermore, the breaking of SIS in *cis*-like configurations, results in the formation of two inequivalent edges, each terminated by a distinct ligand. As a result, in these configurations the calculated edge states differ between the left and right edges. These distinct edge states, referred to as valley-edge states, are hallmark features of the VHE in noncentrosymmetric structures.^55^

The semi-infinite edge states and band structures of 1D nanoribbons —with widths as large as 40-unit cells—were calculated for *cis*-like configurations. Figure S11 presents the valley-edge states and the corresponding BC distributions in the (*k*_*x*_, *k*_*y*_) plane, for six *cis*-like configurations, with two electrons doped per unit cell. As previously discussed, the II­(NiS_2_O_2_)_c_^–2^ and III­(NiSe_2_O_2_)_c_^–2^ configurations, characterized by the largest anionic inner potential difference and largest Dirac band gap, exhibit the most pronounced BC distributions within the BZ. The similar BC distributions are observed in tight-binding studies by changing the signs of the relative hopping parameters in systems incorporating organic ligands^54^ and edge states similar to those observed in these two configurations have been identified in other structures exhibiting the VHE.^75–77^

The position of the edge states at the left or right boundary is clearly influenced by the boundary geometry. For instance, in II­(NiS_2_Se_2_)_c_^–2^ the position of the Se atoms determine the edge states position (see Figure S12). These findings indicate that, in I­(NiO_2_S_2_)_c_^–2^, I­(NiO_2_Se_2_)_c_^–2^, II­(NiS_2_O_2_)_c_^–2^, III­(NiSe_2_O_2_)_c_^–2^ and III­(NiSe_2_S_2_)_c_^–2^, the edge states predominantly localize on the boundary, where the terminating atoms involve S and Se atoms. This behavior may be attributed to the lower electronegativity and more delocalized charge distribution of Se compared to S and O, which facilitates greater orbital overlap with neighboring atoms. Similarly, S atoms exhibit a more delocalized charge distribution than O, further influencing the location of the edge states. The boundary geometry of the I­(NiO_2_Se_2_)_c_^–2^ nanoribbon is illustrated in Figure S13.

An intriguing observation is that, in the II­(NiS_2_O_2_)_c_^–2^ and III­(NiSe_2_O_2_)_c_^–2^ configurations, the edge states are significantly different and exclusively localized on the side terminated by S and Se, as indicated by the red bands, while the blue bands—corresponding to the opposite edge—are absent at the Fermi level. This behavior stands in contrast to the other four configurations, which exhibit edge states on both sides. A plausible explanation for this difference is the presence of significant tensile tension, which promotes electron localization and diminishes orbital overlap on the oxygen-terminated edge.

Figure S14 illustrates the distribution of the BC at the VB for six *cis*-like configurations in the absence of SOC. Notably, the BC of the occupied bands becomes nonzero even in the absence of SOC, except for the III­(NiSe_2_O_2_)_c_^–2^ configuration. This exception can be attributed to its pronounced potential difference, which is the largest among these six *cis*-like configurations. These results indicate that induced VHE in these structures is independent of SOC. Such behavior contrasts with that observed in certain transition metal dichalcogenides, where SOC plays a crucial role in generating the VHE.^78^

### 3.3 Topological Properties of *Trans*-Like Configurations

Following the investigation of the topological properties of *cis*-like configurations, this section focuses on the *trans*-like configurations. Figure 7 presents the intrinsic contribution to the SHC, where the spin Hall current is driven by the SBC of the Bloch bands in six *trans*-like configurations including I­(NiO_2_S_2_)_t_^–2^, I­(NiO_2_Se_2_)_t_^–2^, II­(NiS_2_O_2_)_t_^–2^, II­(NiS_2_Se_2_)_t_^–2^, III­(NiSe_2_O_2_)_t_^–2^ and III­(NiSe_2_S_2_)_t_^–2^ structures.

**Figure 7:**
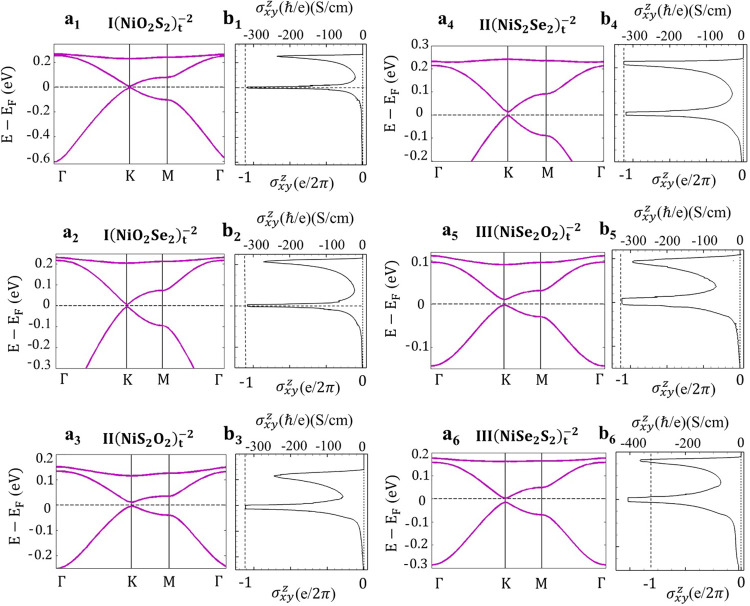
(a_1_–a_6_) Relativistic band structures of six reduced-symmetry structures with *trans*-like configurations: I­(NiO_2_S_2_)_t_^–2^, I­(NiO_2_Se_2_)_t_^–2^, II­(NiS_2_O_2_)_t_^–2^, II­(NiS_2_Se_2_)_t_^–2^, III­(NiSe_2_O_2_)_t_^–2^, and III­(NiSe_2_S_2_)_t_^–2^, respectively. The purple bands represent Kagome bands. (b_1_–b_6_) Corresponding SHCs with Fermi energy scan.

The pristine I­(NiO_4_),^79^ II­(NiS_4_),^35^ and III­(NiSe_4_)^79^ structures exhibit maximal SHC when the Fermi level is shifted into the Dirac band gap via two-electron doping. The *trans*-like configurations display analogous behavior, with an enhancement of the SHC observed under similar doping conditions. Notably, in one of the six configurations—specifically II­(NiS_2_Se_2_)_t_^–2^—the quantization of the SHC is preserved within both nontrivial gaps.^35^ In contrast, for configurations incorporating oxygen ligands—such as I­(NiO_2_S_2_)_t_^–2^, I­(NiO_2_Se_2_)_t_^–2^, II­(NiS_2_O_2_)_t_^–2^ and III­(NiSe_2_O_2_)_t_^–2^, the SHC remains quantized within the Dirac band gap but diminishes in the secondary band gap and resulting in a nonquantized response. The secondary band gap in these structures is a local gap between the nearly FB and the upper Dirac band, which can be reached, for example, by a four-electron doping concentration (see Figure S8e). This phenomenon can be attributed to the closure of the global band gap in these regions, resembling the behavior observed in the pristine I­(NiO_4_).^79^

The removal of the global gap gives rise to a nontrivial topological phase known as the Z_2_ metallic phase.^80^ This phase is so named because, despite exhibiting metallic behavior and lacking a global band gap, the system retains a nonzero topological invariant (Z_2_ = 1). Figure 7 illustrates the band structures, for the six *trans*-like configurations studied under two-electron doping concentrations, along with their corresponding SHC, σ_*xy*_^*z*^, as a function of Fermi energy. Note that because of their symmetry constraints^81^ and of their 2D structure, only the σ_*xy*_^*z*^ component of the SHC tensor is nonzero. The appearance of the Z_2_ metallic phase in *trans*-like configurations can be attributed to the presence of O atoms, which facilitate next-nearest neighbor hopping.^80^ Specifically, the I­(NiO_2_S_2_)_t_^–2^, I­(NiO_2_Se_2_)_t_^–2^, II­(NiS_2_O_2_)_t_^–2^ and III­(NiSe_2_O_2_)_t_^–2^ configurations are capable of hosting this nontrivial phase at high electron doping concentrations, when the Fermi level is elevated into the region between the upper Dirac band and the nearly FB.

Finally, compared to the other five *trans*-like configurations, III­(NiSe_2_S_2_)_t_^–2^ exhibits a distinct response, displaying nonquantized SHC at both nontrivial gaps, with nonequal values. Figure 7b_6_ shows that the SHC reduces in the second nontrivial gap of III­(NiSe_2_S_2_)_t_^–2^ located near 0.2 eV, because this gap is substantially smaller than the first topological gap at the Fermi level (see Figure S7c_7_). So, the SHC plateau width, and also its magnitude, is strongly correlated with the topological gap size. This finite remained gap, allows the SHC to maintain a tinny plateau and preventing a transition to a Z_2_ metallic phase, unlike the other four *trans*-like configurations. While in the II­(NiS_2_Se_2_)_t_^–2^, the two nontrivial gaps have nearly similar magnitudes, resulting in almost equal SHC values and plateau width, in both topological gaps (see Figure 7b_4_).

In an ideal quantum spin Hall (QSH) insulator with conserved spin , the SHC is expected to be quantized as $\sigma_{xy}^{z}=n\frac{e}{2\pi }$ , where *n* is an integer.^82^ However, in realistic QSH materials, the SHC is often not perfectly quantized. Previous studies have shown that, even systems with a nontrivial topological invariant, *Z*_2_ = 1, may exhibit substantial deviations from the ideal quantized value.^83^ The quantization is influenced by various factors, including crystal symmetry^84,85^ and external parameters such as mirror symmetry breaking induced by an electric field or substrate,^82^ as well as biaxial strain.^84^ Furthermore, the nonconserved is destructive for quantized SHC.^85^ These results indicate that a QSH phase is not universally characterized by quantized SHC.^84^ For the present systems, the deviation of the SHC from –1*e*/2π originates from the combined effects of: (i) reduced crystal symmetry induced by strain, and (ii) differences in the internal electrostatic potential between two structures possessing the same ligands, where one exhibits quantized SHC, II­(NiS_2_Se_2_)_t_^–2^, while the other displays nonquantized SHC, III­(NiSe_2_S_2_)_t_^–2^.

The internal electrostatic potential profiles shown in Figure S15 provide microscopic insight into the observed SHC deviation. The structure II­(NiS_2_Se_2_)_t_^–2^ exhibits a deeper and more spatially varying potential profile than III­(NiSe_2_S_2_)_t_^–2^, particularly in the central region of the unit cell. Such variations in the internal potential modify the electronic localization and orbital hybridization, thereby influencing the effective SOC strength and the degree of spin mixing (see Figure S16d_4_ and d_6_ for SBC) in the topological states. Since quantized SHC requires approximate conservation of , the enhanced spin mixing associated with stronger potential modulation can lead to deviations of the SHC from the ideal quantized value. The accompanying differences in charge density further support the existence of distinct electronic environments in the two systems, which contribute to their different SHC responses.

Figure S16 presents the helical edge states of the all *trans*-like configurations, at the Fermi level within their nontrivial band gaps. The sign of σ_*xy*_^*z*^ depends on the SBC sign, and the emergence of negative SBC at the VB results in negative peaks in σ_*xy*_^*z*^, see Figure S16. Despite the reduction in symmetry, most of these *trans*-like configurations preserve their nontrivial topological character exhibiting helical edge states, a nonzero topological invariant, and quantized SHC. In these systems, the helical edge states are present on both sides of the semi-infinite structures, similar to the pristine ones.

### 3.4 Topological Properties of Nonpristine Homogeneous Structures

Finally, we investigated the topological properties of six I, II and III­(NiX_2_Y_2_) structures with X = Y = O, S or Se, obtained by substituting all organic ligands with other iso-valent counterparts. The resulting configurations include only nonpristine homogeneous structures I­(NiS_4_), I­(NiSe_4_), II­(NiO_4_), II­(NiSe_4_), III­(NiO_4_) and III­(NiS_4_). Figure S17 present the band structures of 1D semi-infinite nonpristine homogeneous configurations and corresponding 2D distribution of the SBC in the (*k*_*x*_ *, k*_*y*_) plane. Among these structures, only II­(NiO_4_) and III­(NiO_4_) cannot preserve their nontrivial topological phases, which is attributed to the maximum tensile tension induced by ligand substitution and the resulting shifting and merging of the Dirac points. The shift of the Dirac point has also been reported in other studies.^21,86^

## 4 Conclusions

In the present study, various topological phases were engineered by manipulating the geometrical symmetry of three nickel-based MOFs incorporating O, S, and Se ligands, Ni_3_C_12_O_12_, Ni_3_C_12_S_12_ and Ni_3_C_12_Se_12_, respectively. Through systematic substitution of the iso-valent ligands (O, S and Se), 18 distinct structures exhibiting diverse symmetries, were successfully constructed.

Among the 18 engineered structures, six adopted a *cis*-like configuration, wherein half of the host ligands were substituted with iso-valent guest ligands, leading to the breaking of SIS. Another six structures exhibited a *trans*-like configuration, involving a substitution of the half of host ligands, which disrupted specific mirror symmetries within the framework. The remaining six structures were homogeneous, with complete replacement of host ligands by iso-valent guest ligands, preserving the overall symmetry of the original framework.

The substitution of ligands with varying atomic radii introduces structural tension within the frameworks, manifesting as either tensile or compressive tension. This tension plays a pivotal role in modulating the electronic properties of the materials, particularly affecting the bandwidth of the Kagome bands. Notably, compressive tension leads to an increase in bandwidth, while tensile tension causes a decrease, underscoring the sensitivity of electronic structure to lattice distortions induced by ligand substitution.

Furthermore, manipulation of geometrical symmetry has a profound impact on the topological characteristics of the studied MOFs. In particular, SIS breaking in the *cis*-like configurations triggers transition from a two-dimensional topological insulator (2D-TI) phase to phenomena associated with the VHE, a type of Hall effect that emerges due to SIS breaking and survives in time-reversal invariant systems. The observed variations in valley edge states and the distribution of the BC across the six *cis*-like configurations were primarily attributed to differences in the anionic inner potential, highlighting the sensitivity of topological features to subtle changes in ligand-induced electrostatic environments.

Although the *trans*-like configurations initially retained the 2D-TI phase at two-electron doping concentrations, their topological behavior diverged as the doping level increased. Notably, systems incorporating O ligands—either as host or guest species—exhibited distinct topological behaviors compared to the O free structures. Similar to the pristine Ni_3_C_12_O_12_ MOF, several *trans*-like configurations containing O ligands—namely I­(NiO_2_S_2_)_t_^–2^, I­(NiO_2_Se_2_)_t_^–2^, II­(NiS_2_O_2_)_t_^–2^ and III­(NiSe_2_O_2_)_t_^–2^—undergo a transition to a Z_2_ metallic phase at high electron doping levels. In contrast, the II­(NiS_2_Se_2_)_t_^–2^ preserves its nontrivial topological character and maintains a quantized SHC, even at high electron doping concentrations. Meanwhile, III­(NiSe_2_S_2_)_t_^–2^ deviates from quantized SHC at both electron doping levels, while retaining its nontrivial topological edge states and Z_2_ invariant. Overall, in *trans*-like configurations, charge-to-spin conversion is tuned through symmetry manipulation.

Finally, among the six nonpristine homogeneous structures—each featuring complete substitution of all 12 ligands—the 2D-TI phase was preserved in I­(NiS_4_), I­(NiSe_4_), II­(NiSe_4_) and III­(NiS_4_). These configurations retain the Dirac point, in contrast to the other structures. In structures containing guest oxygen ligands—II­(NiO_4_) and III­(NiO_4_)—which subjected to large tensile tension the Dirac points shift away from their original positions (***K*** and ***K*** ′), get merged and a trivial insulating phase emerges. This behavior indicates that lattice strain plays a crucial role in maintaining topological protection.

Finally, to systematically compare these effects across all examined structures, a comprehensive summary of the topological and electronic characteristics of all 18 configurations is presented in Table 2.

**Table 2:**
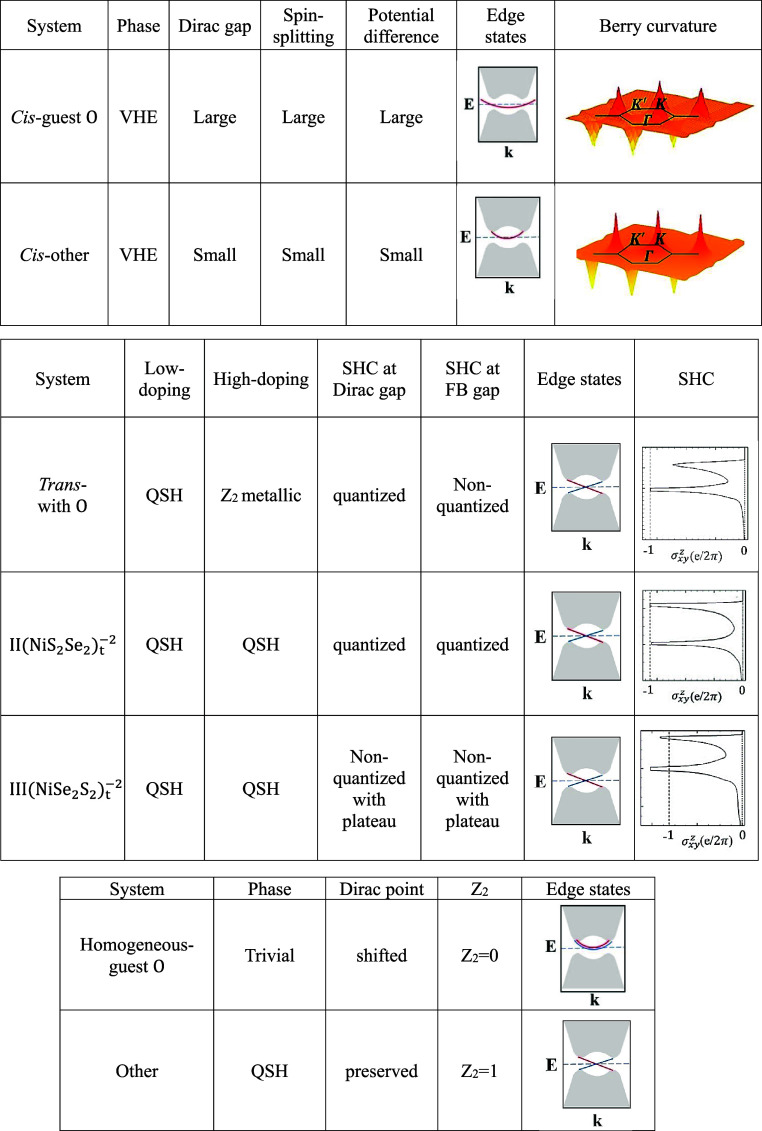
Quick Overview of All Findings from This Study, Illustrating Topological and Electronic Properties of *cis*-Like, *trans*-Like, and Homogeneous Configurations

## Supplementary Material



## Notes

Crystal structures and band structures with and without SOC for twenty-one configurations, cohesive energies (*E*_C_) for pristine structures without and under electron doping; a summary of three types of SOC band gaps for all configurations, Bader charge analyses of the studied systems; semi-infinite band structures of *trans*-like, *cis*-like, and homogeneous configurations; and Berry curvature (BC) of *cis*-like configurations calculated without SOC (PDF)

## References

[1] Hirsch J. E. (1999). Spin Hall
Effect. Phys. Rev. Lett..

[2] Kondou K., Otani Y. (2023). Emergence of Spin–Charge Conversion
Functionalities Due to
Spatial and Time-Reversal Asymmetries and Chiral Symmetry. Front. Phys..

[3] Wang Z. F., Su N., Liu F. (2013). Prediction
of a two-dimensional organic topological
insulator. Nano Lett..

[4] Wang Z. F., Liu Z., Liu F. (2013). Organic topological
insulators in organometallic lattices. Nat.
Commun..

[5] Wang Z. F., Liu Z., Liu F. (2013). Quantum anomalous
Hall effect in 2D organic topological
insulators. Phys. Rev. Lett..

[6] Huang Z., Geilhufe R. M. (2024). Quantum Metal-Organic
Frameworks. Small Sci..

[7] Liu Z., Wang Z. F., Mei J. W., Wu Y. S., Liu F. (2013). Flat Chern
band in a two-dimensional organometallic framework. Phys. Rev. Lett..

[8] Dong L., Kim Y., Er D., Rappe A. M., Shenoy V. B. (2016). Two-Dimensional
π-Conjugated Covalent-Organic Frameworks as Quantum Anomalous
Hall Topological Insulators. Phys. Rev. Lett..

[9] Yamada M. G., Soejima T., Tsuji N., Hirai D., Dincă M., Aoki H. (2016). First-Principles Design
of a Half-Filled Flat Band of the Kagome
Lattice in Two-Dimensional Metal-Organic Frameworks. Phys. Rev. B.

[10] Zhou Q., Wang J., Chwee T. S., Wu G., Wang X., Ye Q., Xu J., Yang S. W. (2015). Topological Insulators Based on 2D
Shape-Persistent Organic Ligand Complexes. Nanoscale.

[11] Ni X., Li H., Liu F., Brédas J. L. (2022). Engineering of Flat Bands and Dirac
Bands in Two-Dimensional Covalent Organic Frameworks (COFS): Relationships
Among Molecular Orbital Symmetry, Lattice Symmetry, and Electronic-Structure
Characteristics. Mater. Horiz..

[12] Xiao D., Liu G. B., Feng W., Xu X., Yao W. (2012). Coupled Spin
and Valley Physics in Monolayers of MoS_2_ and other Group-VI
Dichalcogenides. Phys. Rev. Lett..

[13] Tao L. L., Tsymbal E. Y. (2019). Two-Dimensional
Spin-Valley Locking Spin Valve. Phys. Rev. B.

[14] Zhou S. Y., Gweon G. H., Fedorov A. V., First P. D., De Heer W. A., Lee D. H., Guinea F., Castro Neto A. H., Lanzara A. (2007). Substrate-Induced Bandgap Opening
in Epitaxial Graphene. Nat. Mater..

[15] Gwo S., Shih C. K. (1993). Site-Selective Imaging
in Scanning Tunneling Microscopy
of Graphite: the Nature of Site Asymmetry. Phys.
Rev. B.

[16] Liu C. C., Zhou J. J., Yao Y. (2015). Valley-Polarized
Quantum Anomalous
Hall Phases and Tunable Topological Phase Transitions in Half-Hydrogenated
Bi Honeycomb Monolayers. Phys. Rev. B.

[17] Zhai X., Blanter Y. M. (2020). Topological Valley
Transport of Gapped Dirac Magnons
in Bilayer Ferromagnetic Insulators. Phys. Rev.
B.

[18] Chen H., Zhou P., Liu J., Qiao J., Oezyilmaz B., Martin J. (2020). Gate Controlled Valley
Polarizer in Bilayer Graphene. Nat. Commun..

[19] Liu L., Zhao B., Zhang J., Bao H., Huan H., Xue Y., Li Y., Yang Z. (2021). Prediction of Coexistence of Anomalous
Valley Hall and Quantum Anomalous Hall Effects in Breathing Kagome-Honeycomb
Lattices. Phys. Rev. B.

[20] Du W., Peng R., He Z., Dai Y., Huang B., Ma Y. (2022). Anomalous Valley Hall Effect in Antiferromagnetic Monolayers. npj 2D Mater. Appl..

[21] Serbyn M., Fu L. (2014). Symmetry breaking and
Landau quantization in topological crystalline
insulators. Phys. Rev. B.

[22] Mak K. F., McGill K. L., Park J., McEuen P. L. (2014). The Valley Hall
Effect in MoS_2_ Transistors. Science.

[23] Ominato Y., Fujimoto J., Matsuo M. (2020). Valley-Dependent
Spin Transport in
Monolayer Transition-Metal Dichalcogenides. Phys. Rev. Lett..

[24] Zeng H., Dai J., Yao W., Xiao D., Cui X. (2012). Valley Polarization
in MoS_2_ Monolayers by Optical Pumping. Nat. Nanotechnol..

[25] Abdollahi M., Bagheri Tagani M. (2020). Tuning Intrinsic Ferromagnetic and Anisotropic Properties
of the Janus VSeS Monolayer. J. Mater. Chem.
C.

[26] Li R., Cheng Y., Huang W. (2018). Recent Progress
of Janus 2D Transition
Metal Chalcogenides: from Theory to Experiments. Small.

[27] Lu A. Y., Zhu H., Xiao J., Chuu C. P., Han Y., Chiu M. H., Cheng C. C., Yang C. W., Wei K. H., Yang Y., Wang Y., Sokaras D., Nordlund D., Yang P., Muller D. A., Chou M. Y., Zhang X., Li L. J. (2017). Janus Monolayers
of Transition Metal Dichalcogenides. Nat. Nanotechnol..

[28] Jang C. W., Lee W. J., Kim J. K., Park S. M., Kim S., Choi S. H. (2022). Growth of Two-Dimensional Janus MoSSe by a Single in
Situ Process without Initial or Follow-Up Treatments. NPG Asia Mater..

[29] Zhang J., Jia S., Kholmanov I., Dong L., Er D., Chen W., Guo H., Jin Z., Shenoy V. B., Shi L., Lou J. (2017). Janus Monolayer
Transition-Metal Dichalcogenides. ACS Nano.

[30] Hu T., Jia F., Zhao G., Wu J., Stroppa A., Ren W. (2018). Intrinsic
and Anisotropic Rashba Spin Splitting in Janus Transition-Metal Dichalcogenide
Monolayers. Phys. Rev. B.

[31] Babaee T. S., Ghobadi N. (2021). Structural, Electrical, and Rashba
Properties of Monolayer
Janus Si_2_XY (X, Y = P, As, Sb, and Bi). Phys. Rev. B.

[32] Zhao J., Qi Y., Yao C., Zeng H. (2024). Tunable Valley-Spin Splitting in
a Janus XMSiN_2_ Monolayer (X= S, Se; M= Mo, Cr) and Giant
Valley Polarization via Vanadium Doping. Phys.
Rev. B.

[33] Dresselhaus G. (1955). Spin-Orbit
Coupling Effects in Zinc Blende Structures. Phys. Rev..

[34] Bychkov Y. A., Rashba É.I. (1984). Properties
of a 2D Electron Gas with Lifted Spectral
Degeneracy. JETP Lett..

[35] Falsafi N., Abedinpour S. H., Nazari F., Illas F. (2025). Tuning Topologically
Nontrivial States in the BHT-Ni Metal–Organic Framework. J. Phys. Chem. C.

[36] Giannozzi P., Andreussi O., Brumme T., Bunau O., Buongiorno
Nardelli M., Calandra M., Car R., Cavazzoni C., Ceresoli D., Cococcioni M., Colonna N., Carnimeo I., Dal Corso A., de Gironcoli S., Delugas P., DiStasio R. A., Ferretti A., Floris A., Fratesi G., Fugallo G., Gebauer R., Gerstmann U., Giustino F., Gorni T., Jia J., Kawamura M., Ko H. Y., Kokalj A., Küçükbenli E., Lazzeri M., Marsili M., Marzari N., Mauri F., Nguyen N. L., Nguyen H. V., Otero-de-la-Roza A., Paulatto L., Poncé S., Rocca D., Sabatini R., Santra B., Schlipf M., Seitsonen A. P., Smogunov A., Timrov I., Thonhauser T., Umari P., Vast N., Wu X., Baroni S. (2017). Advanced Capabilities
for Materials Modelling with Quantum ESPRESSO. J. Phys.: Condens. Matter.

[37] Perdew J. P., Burke K., Ernzerhof M. (1996). Generalized
Gradient Approximation
Made Simple. Phys. Rev. Lett..

[38] Kang S., Yu J. (2022). Electronic Structure
and Magnetic Properties of Transition Metal
Kagome Metal–Organic Frameworks. Phys.
Chem. Chem. Phys..

[39] He T., Zhang X., Li Y., Jin L., Liu Y., Liu G., Yuan H. (2023). Metal-Organic Framework as High-Order Topological Insulator
with Protected Corner Modes. Mater. Today Nano.

[40] Crasto
de Lima F., Ferreira G. J., Miwa R. H. (2019). Layertronic control
of topological states in multilayer metal-organic frameworks. J. Chem. Phys..

[41] Kresse G., Joubert D. (1999). From Ultrasoft Pseudopotentials
to the Projector Augmented-Wave
Method. Phys. Rev. B.

[42] Mostofi A. A., Yates J. R., Pizzi G., Lee Y.-S., Souza I., Vanderbilt D., Marzari N. (2014). An Updated Version
of Wannier90:
A Tool for Obtaining Maximally-Localised Wannier Functions. Comput. Mater. Sci..

[43] Qiao J., Zhou J., Yuan Z., Zhao W. (2018). Calculation
of Intrinsic
Spin Hall Conductivity by Wannier Interpolation. Phys. Rev. B.

[44] Bercioux, D. ; Cayssol, J. ; Vergniory, M. G. ; Calvo, M. R. Topological Matter: Lectures from the Topological Matter School 2017. Springer, 2018.

[45] Wu Q. S., Zhang S. N., Song H.-F., Troyer M., Soluyanov A. A. (2018). WANNIERTOOLS:
An Open-Source Software Package for Novel Topological Materials. Comput. Phys. Commun..

[46] Gradhand M., Fedorov D. V., Pientka F., Zahn P., Mertig I., Györffy B. L. (2012). First-Principle Calculations of the
Berry Curvature
of Bloch States for Charge and Spin Transport of Electrons. J. Phys.: Condens. Matter.

[47] Thouless D. J., Kohmoto M., Nightingale M. P., den Nijs M. (1982). Quantized Hall Conductance
in a Two-Dimensional Periodic Potential. Phys.
Rev. Lett..

[48] Kambe T., Sakamoto R., Hoshiko K., Takada K., Miyachi M., Ryu J. H., Sasaki S., Kim J., Nakazato K., Takata M., Nishihara H. (2013). π-Conjugated
Nickel Bis (Dithiolene)
Complex Nanosheet. J. Am. Chem. Soc..

[49] Mortazavi B., Shahrokhi M., Hussain T., Zhuang X., Rabczuk T. (2019). Theoretical
Realization of Two-Dimensional M_3_(C_6_ X_6_)_2_ (M= Co, Cr, Cu, Fe, Mn, Ni, Pd, Rh and X= O, S, Se)
Metal–Organic Frameworks. Appl. Mater.
Today.

[50] Deng T., Shi W., Wong Z. M., Wu G., Yang X., Zheng J. C., Pan H., Yang S. W. (2021). Designing Intrinsic Topological Insulators in Two-Dimensional
Metal–Organic Frameworks. J. Phys. Chem.
Lett..

[51] Hambley T. W. (2001). Platinum
binding to DNA: structural controls and consequences. J. Chem. Soc., Dalton Trans..

[52] Wilson J. J., Lippard S. J. (2014). Synthetic methods for the preparation of platinum anticancer
complexes. Chem. Rev..

[53] Barreteau C., Ducastelle F., Mallah T. (2017). A Bird’s Eye View on the Flat
and Conic Band World of the Honeycomb and Kagome Lattices: Towards
an Understanding of 2D Metal-Organic Frameworks Electronic Structure. J. Condens. Matter Phys..

[54] Shuku Y., Suizu R., Nakano S., Tsuchiizu M., Awaga K. (2023). Engineering Dirac Cones and Topological Flat Bands with Organic Molecules. Phys. Rev. B.

[55] Wang Y., Wang H. X., Liang L., Zhu W., Fan L., Lin Z. K., Li F., Zhang X., Luan P. G., Poo Y., Jiang J. H., Guo G. Y. (2023). Hybrid Topological Photonic Crystals. Nat. Commun..

[56] Bader R. F. W. (1985). Atom
in Molecules. Acc. Chem. Res..

[57] Bader R. F. W. (1991). A Quantum
Theory of Molecular Structure and its Applications. Chem. Rev..

[58] Henkelman G., Arnaldsson A., Jónsson H. (2006). A Fast and Robust Algorithm for Bader
Decomposition of Charge Density. Comput. Mater.
Sci..

[59] Yates J. R., Wang X., Vanderbilt D., Souza I. (2007). Spectral and Fermi
Surface Properties from Wannier Interpolation. Phys. Rev. B Condens. Matter.

[60] OpenAI . ChatGPT Image Generation (2025). Retrieved from https://openai.com (accessed 2026–02–10).

[61] Olsen T., Souza I. (2015). Valley Hall Effect in Disordered Monolayer MoS_2_ from First
Principles. Phys. Rev. B.

[62] Feng W., Yao Y., Zhu W., Zhou J., Yao W., Xiao D. (2012). Intrinsic
Spin Hall Effect in Monolayers of Group-VI Dichalcogenides: A First-Principles
Study. Phys. Rev. B.

[63] Kosmider K., González J. W., Fernández-Rossier J. (2013). Large Spin
Splitting
in the Conduction Band of Transition Metal Dichalcogenide Monolayers. Phys. Rev. B.

[64] Zhu Z. Y., Cheng Y. C., Schwingenschlögl U. (2011). Giant Spin-Orbit-Induced
Spin Splitting in Two-Dimensional Transition-Metal Dichalcogenide
Semiconductors. Phys. Rev. B Condens. Matter..

[65] Li C., An Y. (2022). Two-Dimensional Intrinsic
Ferrovalley Janus 2H-VSeS Monolayer with
High Curie Temperature and Robust Valley Polarization. Phys. Rev. Mater..

[66] Luo C., Peng X., Qu J., Zhong J. (2020). Valley degree of freedom
in ferromagnetic Janus monolayer H-VSSe and the asymmetry-based tuning
of the valleytronic properties. Phys. Rev. B.

[67] Zhang J., Zhao B., Xue Y., Zhou T., Yang Z. (2018). Coupling Effect
of Topological States and Chern Insulators in Two-Dimensional Triangular
Lattices. Phys. Rev. B.

[68] Zhang J., Zhao B., Ma C., Yang Z. (2019). Prediction of Intrinsic
Two-Dimensional Non-Dirac Topological Insulators in Triangular Metal-Organic
Frameworks. Appl. Phys. Lett..

[69] Li J., Gu L., Wu R. (2020). Transition-Metal Phthalocyanine Monolayers as New Chern
Insulators. Nanoscale.

[70] de
Lima F. C., Ferreira G. J., Miwa R. H. (2017). Tuning the Topological
States in Metal-Organic Bilayers. Phys. Rev.
B.

[71] Vanderbilt, D. Berry Phases in Electronic Structure Theory: Electric Polarization, Cambridge University Press, Orbital Magnetization and Topological Insulators, 2018.

[72] Xiao D., Yao W., Niu Q. (2007). Valley-Contrasting
Physics in Graphene: Magnetic Moment
and Topological Transport. Phys. Rev. Lett..

[73] Tian D., Liu Z., Shen S., Li Z., Zhou Y., Liu H., Chen H., Yu P. (2021). Manipulating
Berry Curvature of SrRuO_3_ Thin Films Via Epitaxial Strain. Proc.
Natl. Acad. Sci. U. S. A..

[74] Sheoran S., Phutela A., Moulik R., Bhattacharya S. (2023). Manipulation
of Valley and Spin Properties in Two-Dimensional Janus WSiGeZ_4_ (Z= N, P, As) through Symmetry Control. J. Phys. Chem. C.

[75] Xi X., Ye K. P., Wu R. X. (2020). Topological Photonic Crystal of Large
Valley Chern Numbers. Photonics Res..

[76] Drouot A., Weinstein M. I. (2020). Edge States
and the Valley Hall Effect. Adv. Math..

[77] Tian Z., Shen C., Li J., Reit E., Bachman H., Socolar J. E. S., Cummer S. A., Jun Huang T. (2020). Dispersion
Tuning and Route Reconfiguration of Acoustic Waves in Valley Topological
Phononic Crystals. Nat. Commun..

[78] Chen X., Zhong L., Li X., Qi J. (2017). Valley Splitting
in
the Transition-Metal Dichalcogenide Monolayer Via Atom Adsorption. Nanoscale.

[79] Falsafi, N. Band Theory Analysis on Electronic and Topological Properties of Two-Dimensional Nickel-Based Metal Organic Frameworks. Ph.D. Thesis, Institute for Advanced Studies in Basic Sciences, 2025.

[80] Zhao B., Zhang J., Feng W., Yao Y., Yang Z. (2014). Quantum Spin
Hall and Z_2_ Metallic States in an Organic Material. Phys. Rev. B.

[81] Roy A., Guimarães M. H., Sławińska J. (2022). Unconventional Spin
Hall Effects in Nonmagnetic Solids. Phys. Rev.
Mater..

[82] Kane C. L., Mele E. J. (2005). Quantum spin Hall
effect in graphene. Phys. Rev. Lett..

[83] Costa M., Schleder G. R., Mera
Acosta C., Padilha A. C. M., Cerasoli F., Buongiorno
Nardelli M., Fazzio A. (2021). Discovery of higher-order topological
insulators using the spin Hall conductivity as a topology signature. Npj Comput. Mater..

[84] Matusalem F., Marques M., Teles L. K., Matthes L., Furthmüller J., Bechstedt F. (2019). Quantization
of spin Hall conductivity in two-dimensional
topological insulators versus symmetry and spin-orbit interaction. Phys. Rev. B.

[85] Zhou J., Poncé S., Charlier J. C. (2025). High-throughput
calculations of spin
Hall conductivity in non-magnetic 2D materials. npj 2D Mater. Appl..

[86] Montambaux G., Piéchon F., Fuchs J. N., Goerbig M. O. (2009). Merging of Dirac
points in a two-dimensional crystal. Phys. Rev.
B Condens. Matter.

