# Supplementary material for: Ligand-Controlled Valley and Spin Properties in Ni-Based Two-Dimensional Metal–Organic Frameworks
Source: J Phys Chem C Nanomater Interfaces. 2026 Jul 4;130(28):9919–31. doi: 10.1021/acs.jpcc.6c01839 (PMC13384018; doi:10.1021/acs.jpcc.6c01839)
Supplement: Supplementary file 1 [file jp6c01839_si_001.pdf]

Supporting Information:

Ligand-Controlled Valley and Spin Properties in Ni-Based Two-Dimensional Metal-Organic Frameworks

Nafiseh Falsafi,[1] Saeed H. Abedinpour,[2] Fariba Nazari*,[1,3] Francesc Illas*[4]

[1]*Department of Chemistry, Institute for Advanced Studies in Basic Sciences, Zanjan 45137-66731, Iran*

[2]*Department of Physics, Institute for Advanced Studies in Basic Sciences, Zanjan 45137-66731, Iran*

[3]*Center of Climate Change and Global Warming, Institute for Advanced Studies in Basic Sciences, Zanjan 45137-66731, Iran*

[4]*Departament de Ciència de Materials i Química Física & Institut de Química Teòrica i Computacional (IQTCUB), Universitat de Barcelona,C/Martí i Franquès 1, 08028 Barcelona, Spain*

Corresponding authors: nazari@iasbs.ac.ir, francesc.illas@ub.edu

CONTENTS:

- **Figure S1**. Band structure of the pristine structures $\mathrm{I(NiO_4)}$, $\mathrm{II(NiS_4)}$ and $\mathrm{III(NiSe_4)}$ calculated for different values of the Hubbard U parameter.
- **Figure S2**. The crystal structures of the twenty-one studied configurations.
- **Figure S3**. Illustrates the SIS breaking in a *cis*-like structure.
- **Table S1**. Cohesive energies (E_C) for pristine structures.
- **Figure S4**. Band structures of the twenty-one configurations, calculated without considering spin–orbit coupling (SOC).
- **Table S2**. Kagome Bands width (BW) at the **Γ** point for the twenty-one configurations.

- **Figure S5**. Projected density of states (PDOS) for the twenty-one configurations.
- **Figure S6**. Bader charge analysis for the twenty-one configurations.
- **Figure S7**. Band structures of the twenty-one configurations.
- **Table S3**. Summary of the three types of band gaps—Dirac, global and local—for all twenty-one configurations.
- **Figure S8**. Band structure of $\mathrm{II(NiS_4)}$ at different electron doping concentrations.
- **Table S4**. Summary of the three types of band gaps—Dirac, global and local—for all twenty-one configurations and under two-electron doping concentration.
- **Table S5**. Cohesive energies (E_C) for pristine structures under two-electron doping concentration.
- **Figure S9**. Relativistic band structure with the projection of the spin operator $\hat{\mathrm{s}}_z$ for the *cis*-like configurations.
- **Figure S10**. Planar electrostatic potential of pristine and *trans*-like configurations.
- **Figure S11**. Valley-edge states and Berry curvature (BC) of one-dimensional semi-infinite *cis*-like configurations.
- **Figure S12**. Edge states of the $\mathrm{II(NiS_2Se_2)_c^{-2}}$ nanoribbon with two distinct terminations, one by sulfur (S) atoms and the other by selenium (Se) atoms.
- **Figure S13**. Nanoribbon geometry and edge states of the $\mathrm{I(NiO_2Se_2)_c^{-2}}$ configuration, showing atomic terminations and corresponding left/right edge-localized states.
- **Figure S14**. Ordinary BC of the VB in *cis*-like configurations along high symmetry lines.
- **Figure S15**. Planar electrostatic potential of two *trans*-like configurations, $\mathrm{II(NiS_2Se_2)_t^{-2}}$ and $\mathrm{III(NiSe_2S_2)_t^{-2}}$ and the corresponding charge density.

- **Figure S16**. Helical edge states and spin Berry curvature (SBC) of 1D semi-infinite *trans*-like configurations.
- **Figure S17**. Edge states and spin Berry curvature (SBC) of 1D semi-infinite homogeneous configurations.

Figure S1. Band structures of the pristine structures $\mathrm{I(NiO_4)}$, $\mathrm{II(NiS_4)}$ and $\mathrm{III(NiSe_4)}$ calculated with different values of the Hubbard U parameter, including U = 0, 3, and 5 eV.

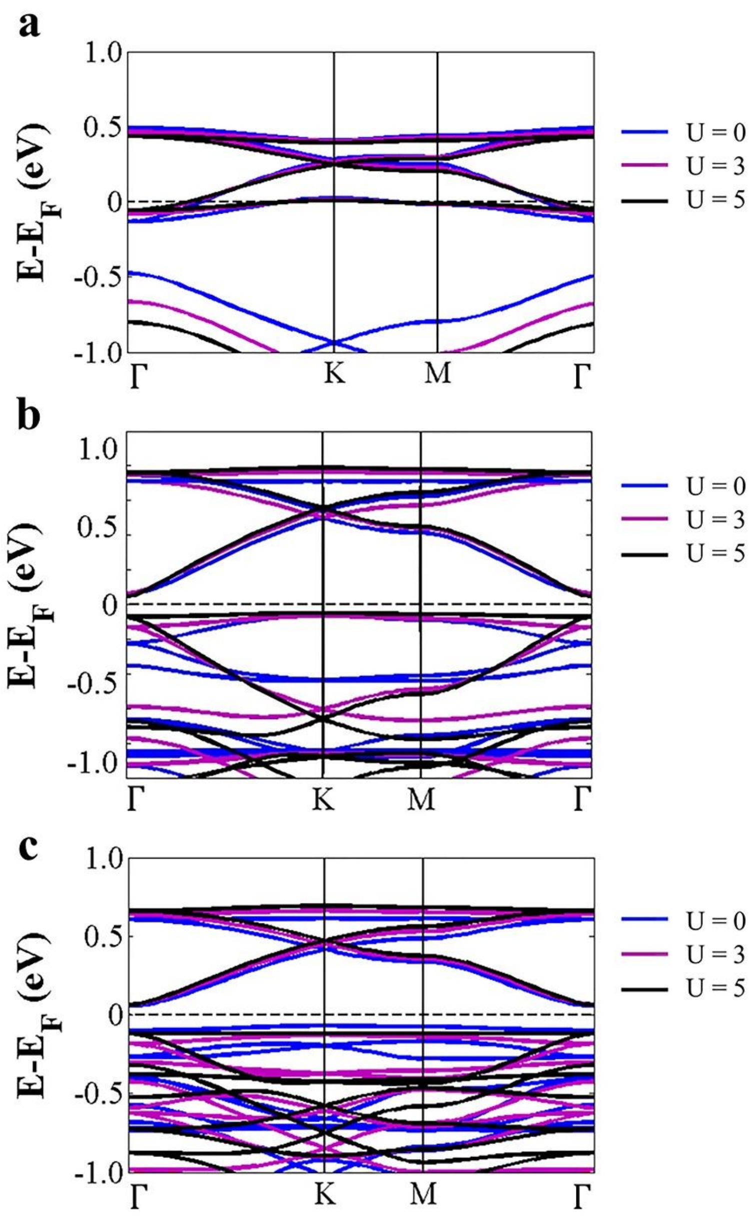

Figure S2. The investigated crystal structures comprise twenty-one configurations, categorized into three groups—Type I, Type II, and Type III—based on the general formula I, II and III(NiX_2Y_2), where X and Y denote O, S or Se atoms arranged in distinct configurations. The first-row presents seven structures with the unit cell area of 146.07 Å², corresponding to Type I. The second-row features structures with the unit cell area of 185.64 Å², classified as Type II. The third-row includes structures with the unit cell area of 204.04 Å², assigned to Type III. The subscripts 'c' and 't' denote *cis*- and *trans*-like configurations, respectively.

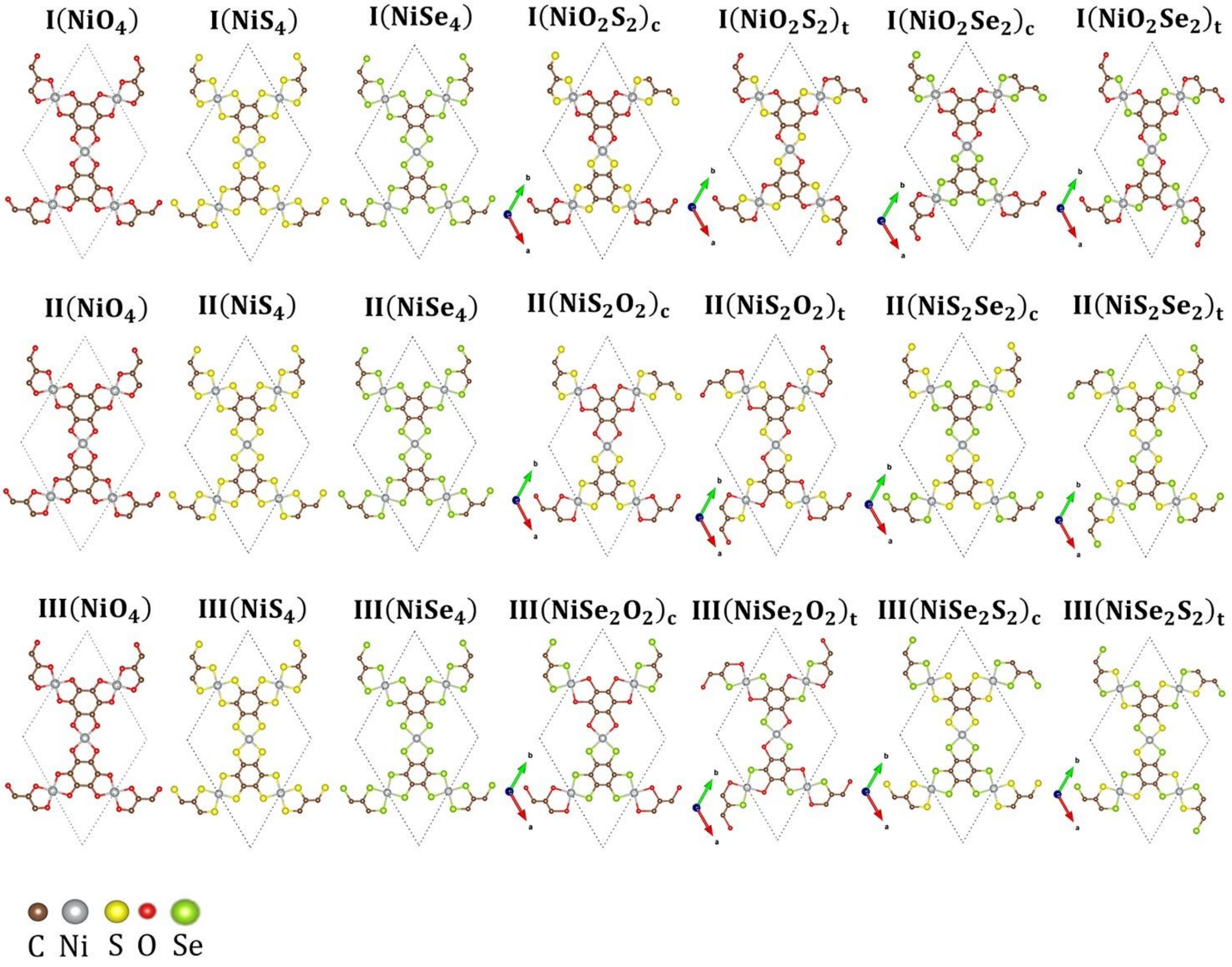

Figure S3. The broken SIS characteristic of *cis*-like structures, is illustrated for $I(NiO_2S_2)_c$, taken as a representative example. The red rings highlight the region where the SIS is breaking. The unit cell is repeated 2×2 for better understanding. Red, yellow, gray and pink circles are O, S, Ni and C atoms, respectively.

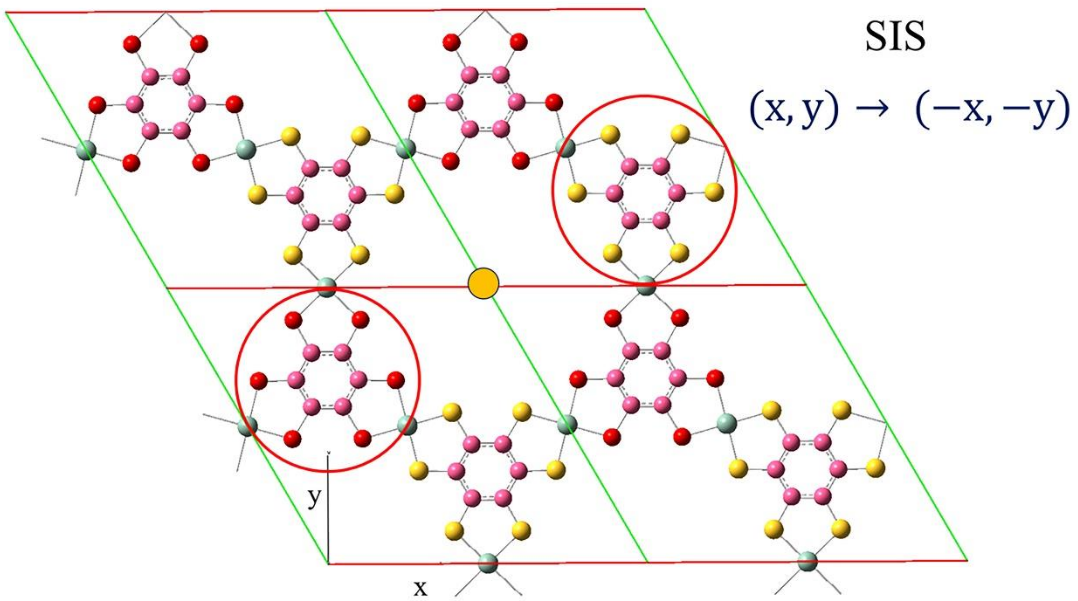

Table S1. Cohesive energies (E_C) per atom (in eV) for pristine structures.

Structure	$I(NiO_4)$	$II(NiS_4)$	$III(NiSe_4)$	graphene
E_C/atom (eV)	-7.41	-6.30	-5.70	-5.65

Figure S4. Band structures of twenty-one configurations, calculated without spin–orbit coupling (SOC), are presented. These include: $\mathrm{I(NiO_4)}$, $\mathrm{I(NiS_4)}$, $\mathrm{I(NiSe_4)}$, $\mathrm{I(NiO_2S_2)_c}$, $\mathrm{I(NiO_2S_2)_t}$, $\mathrm{I(NiO_2Se_2)_c}$, $\mathrm{I(NiO_2Se_2)_t}$ and $\mathrm{II(NiO_4)}$, $\mathrm{II(NiS_4)}$, $\mathrm{II(NiSe_4)}$, $\mathrm{II(NiS_2O_2)_c}$, $\mathrm{II(NiS_2O_2)_t}$, $\mathrm{II(NiS_2Se_2)_c}$, $\mathrm{II(NiS_2Se_2)_t}$ and $\mathrm{III(NiO_4)}$, $\mathrm{III(NiS_4)}$, $\mathrm{III(NiSe_4)}$, $\mathrm{III(NiSe_2O_2)_c}$, $\mathrm{III(NiSe_2O_2)_t}$, $\mathrm{III(NiSe_2S_2)_c}$, $\mathrm{III(NiSe_2S_2)_t}$ structures, respectively. Subscripts ‘c’ and ‘t’ indicate *cis*- and *trans*-like configurations, respectively. Panels (a_1-a_3), (b_1-b_3) and (c_1-c_3) correspond to homogeneous structures. The purple bands correspond to the Kagome bands, while the blue bands represent the other bands.

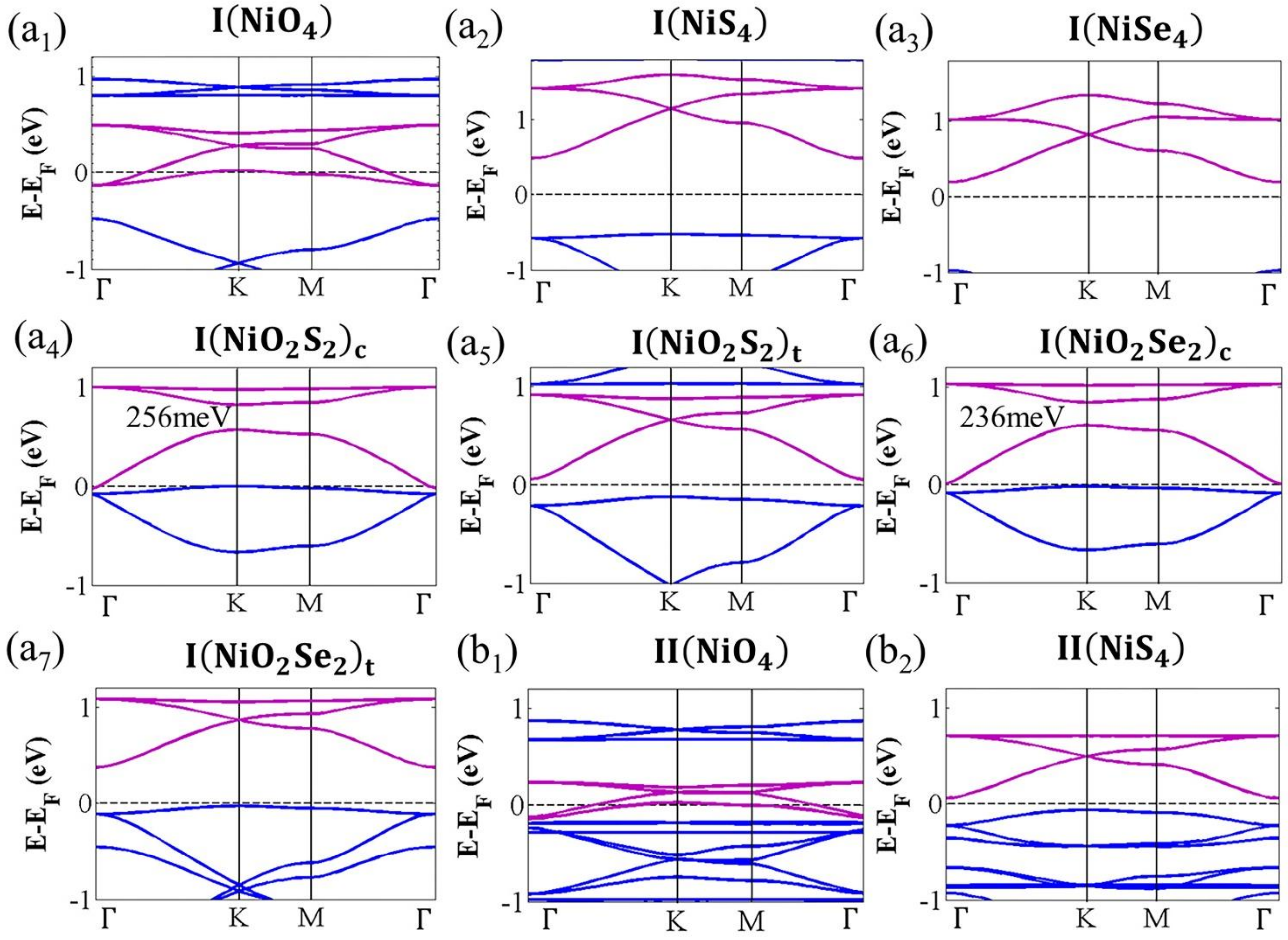

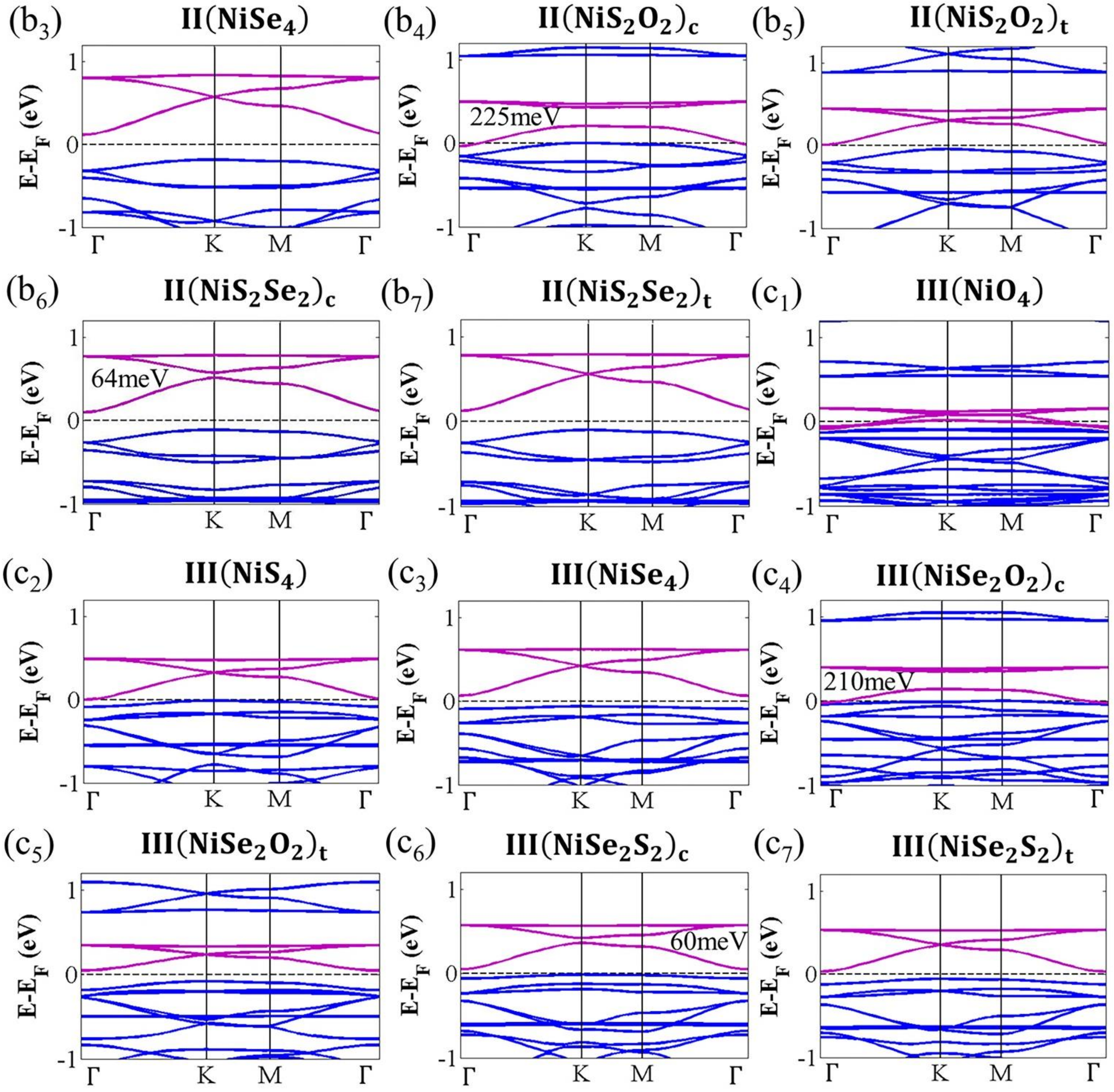
(b3) II(NiSe4)
(b4) II(NiS2O2)c
225meV
(b5) II(NiS2O2)t
(b6) II(NiS2Se2)c
64meV
(b7) II(NiS2Se2)t
(c1) III(NiO4)
(c2) III(NiS4)
(c3) III(NiSe4)
(c4) III(NiSe2O2)c
210meV
(c5) III(NiSe2O2)t
(c6) III(NiSe2S2)c
60meV
(c7) III(NiSe2S2)t
E-EF (eV)
1
0
-1
Γ
K
M
Γ

Table S2: Kagome Bands width (BW) at the **Γ** point for the twenty-one structures, expressed in electron volts (eV).

Structure	$\mathrm{I(NiO_4)}$	$\mathrm{I(NiS_4)}$	$\mathrm{I(NiSe_4)}$	$\mathrm{I(NiO_2S_2)_c}$	$\mathrm{I(NiO_2S_2)_t}$
BW (eV)	0.63	0.92	0.83	1.02	0.86
Structure	$\mathrm{I(NiO_2Se_2)_c}$	$\mathrm{I(NiO_2Se_2)_t}$	$\mathrm{II(NiO_4)}$	$\mathrm{II(NiS_4)}$	$\mathrm{II(NiSe_4)}$
BW (eV)	1.02	0.71	0.42	0.65	0.69
Structure	$\mathrm{II(NiS_2O_2)_c}$	$\mathrm{II(NiS_2O_2)_t}$	$\mathrm{II(NiS_2Se_2)_c}$	$\mathrm{II(NiS_2Se_2)_t}$	$\mathrm{III(NiO_4)}$
BW (eV)	0.53	0.44	0.67	0.65	0.24
Structure	$\mathrm{III(NiS_4)}$	$\mathrm{III(NiSe_4)}$	$\mathrm{III(NiSe_2O_2)_c}$	$\mathrm{III(NiSe_2O_2)_t}$	$\mathrm{III(NiSe_2S_2)_c}$
BW (eV)	0.49	0.55	0.42	0.30	0.52
Structure	$\mathrm{III(NiSe_2S_2)_t}$				
BW (eV)	0.52				

Figure S5. (a_1-c_7) Projected density of states (PDOS) for the twenty-one configurations, calculated without SOC. These include: $\mathrm{I(NiO_4)}$, $\mathrm{I(NiS_4)}$, $\mathrm{I(NiSe_4)}$, $\mathrm{I(NiO_2S_2)_c}$, $\mathrm{I(NiO_2S_2)_t}$, $\mathrm{I(NiO_2Se_2)_c}$, $\mathrm{I(NiO_2Se_2)_t}$ and $\mathrm{II(NiO_4)}$, $\mathrm{II(NiS_4)}$, $\mathrm{II(NiSe_4)}$, $\mathrm{II(NiS_2O_2)_c}$, $\mathrm{II(NiS_2O_2)_t}$, $\mathrm{II(NiS_2Se_2)_c}$, $\mathrm{II(NiS_2Se_2)_t}$ and $\mathrm{III(NiO_4)}$, $\mathrm{III(NiS_4)}$, $\mathrm{III(NiSe_4)}$, $\mathrm{III(NiSe_2O_2)_c}$, $\mathrm{III(NiSe_2O_2)_t}$, $\mathrm{III(NiSe_2S_2)_c}$, $\mathrm{III(NiSe_2S_2)_t}$ structures, respectively. Panels (a_1-a_3), (b_1-b_3) and (c_1-c_3) correspond to homogeneous structures.

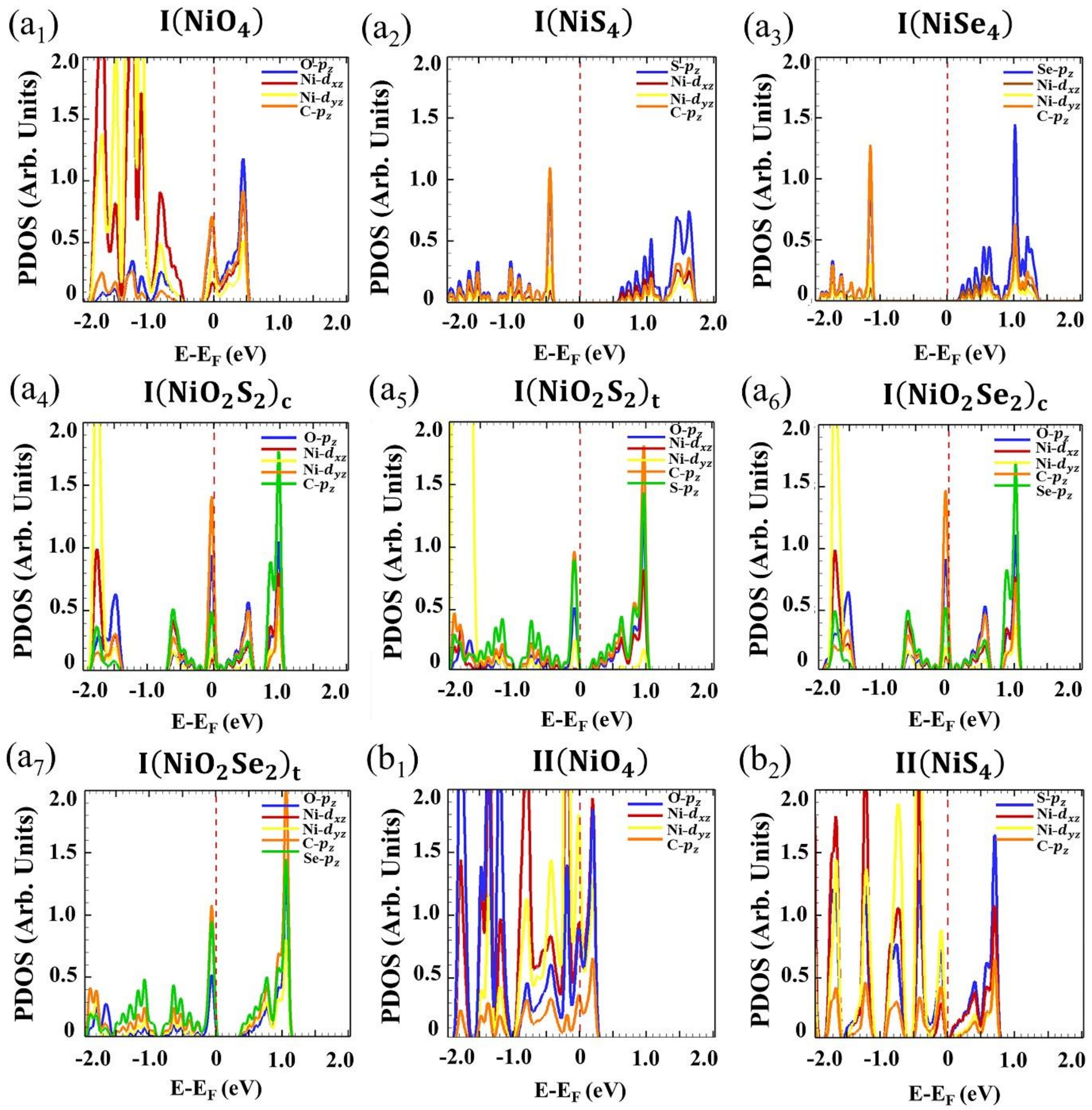

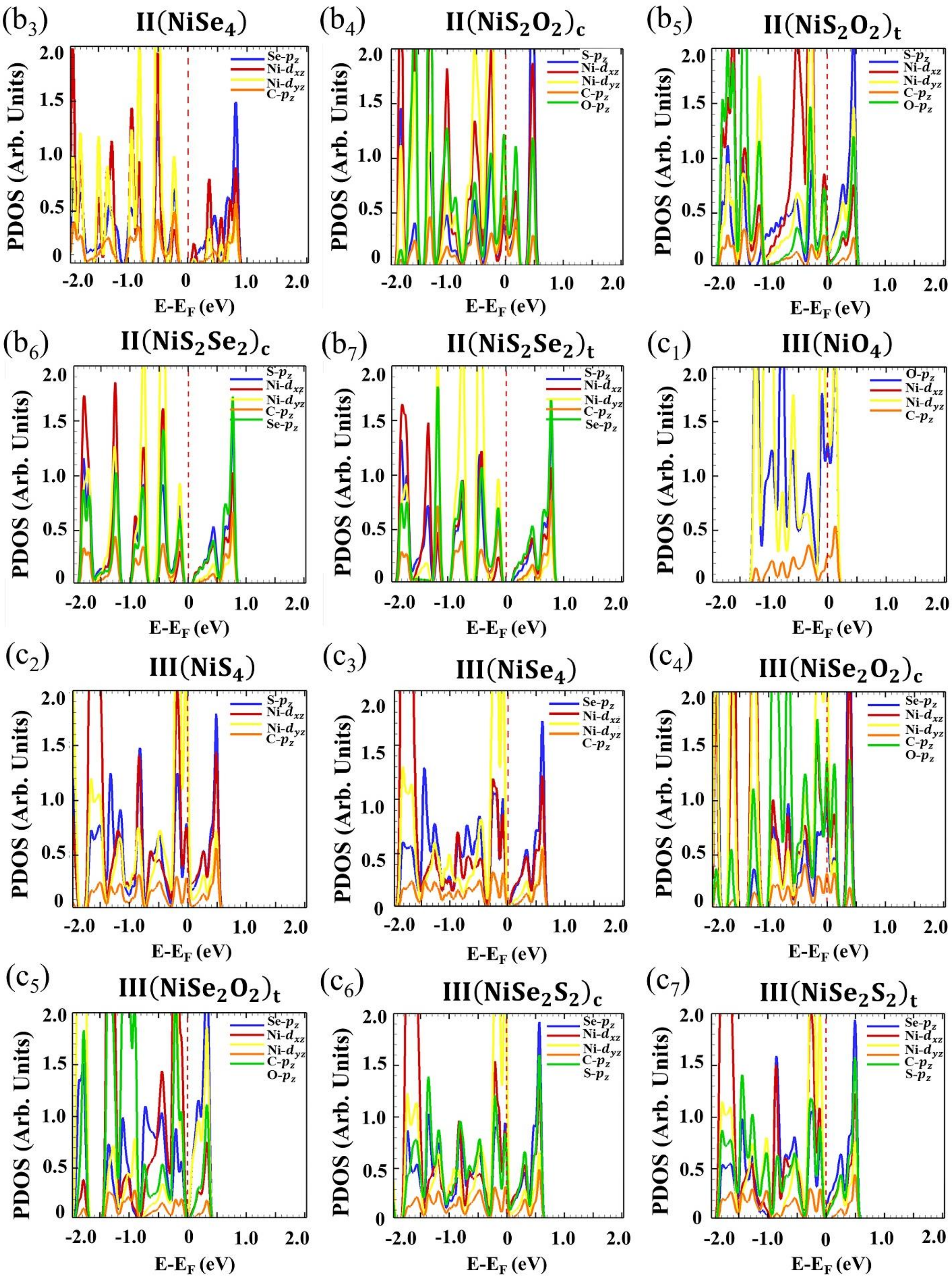
(b3) II(NiSe4)
Se-pz
Ni-dxz
Ni-dyz
C-pz
(b4) II(NiS2O2)c
S-pz
Ni-dxz
Ni-dyz
C-pz
O-pz
(b5) II(NiS2O2)t
S-pz
Ni-dxz
Ni-dyz
C-pz
O-pz
(b6) II(NiS2Se2)c
S-pz
Ni-dxz
Ni-dyz
C-pz
Se-pz
(b7) II(NiS2Se2)t
S-pz
Ni-dxz
Ni-dyz
C-pz
Se-pz
(c1) III(NiO4)
O-pz
Ni-dxz
Ni-dyz
C-pz
(c2) III(NiS4)
S-pz
Ni-dxz
Ni-dyz
C-pz
(c3) III(NiSe4)
Se-pz
Ni-dxz
Ni-dyz
C-pz
(c4) III(NiSe2O2)c
Se-pz
Ni-dxz
Ni-dyz
C-pz
O-pz
(c5) III(NiSe2O2)t
Se-pz
Ni-dxz
Ni-dyz
C-pz
O-pz
(c6) III(NiSe2S2)c
Se-pz
Ni-dxz
Ni-dyz
C-pz
S-pz
(c7) III(NiSe2S2)t
Se-pz
Ni-dxz
Ni-dyz
C-pz
S-pz
PDOS (Arb. Units)
E-EF (eV)
-2.0
-1.0
0
1.0
2.0
0.5
1.5

Figure S6. Bader charge analysis, defined as the deviation between the computed Bader charge and the valence charge of each atom, provides a more accurate assessment of electron gains (negative sign) and losses (positive sign) for each atom.

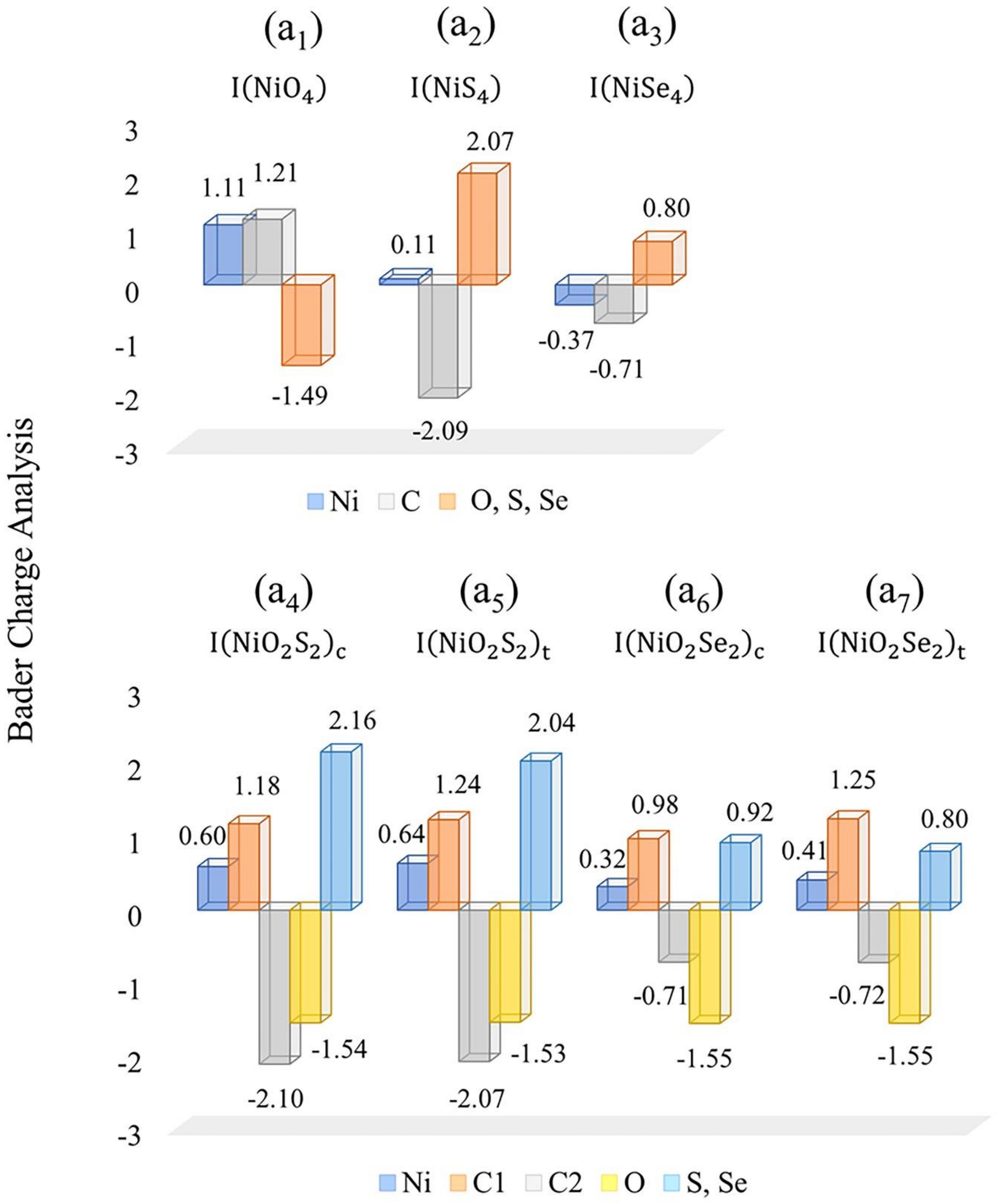

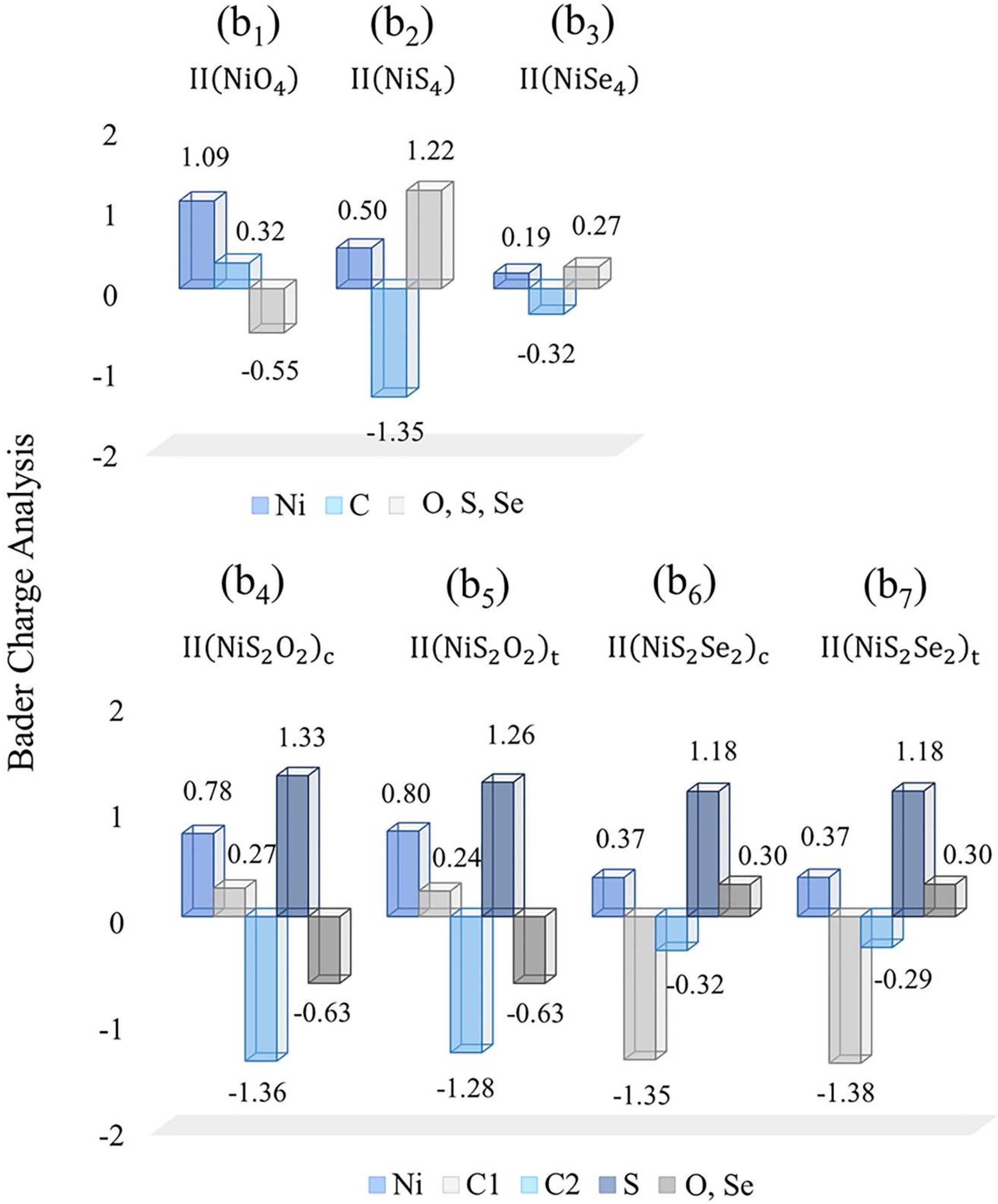
(b1) II(NiO4)
(b2) II(NiS4)
(b3) II(NiSe4)
2
1
0
-1
-2
1.09
0.32
-0.55
0.50
1.22
-1.35
0.19
0.27
-0.32
Ni
C
O, S, Se
Bader Charge Analysis
(b4) II(NiS2O2)c
(b5) II(NiS2O2)t
(b6) II(NiS2Se2)c
(b7) II(NiS2Se2)t
0.78
0.27
1.33
-1.36
-0.63
0.80
0.24
1.26
-1.28
-0.63
0.37
1.18
0.30
-1.35
-0.32
0.37
1.18
0.30
-1.38
-0.29
Ni
C1
C2
S
O, Se

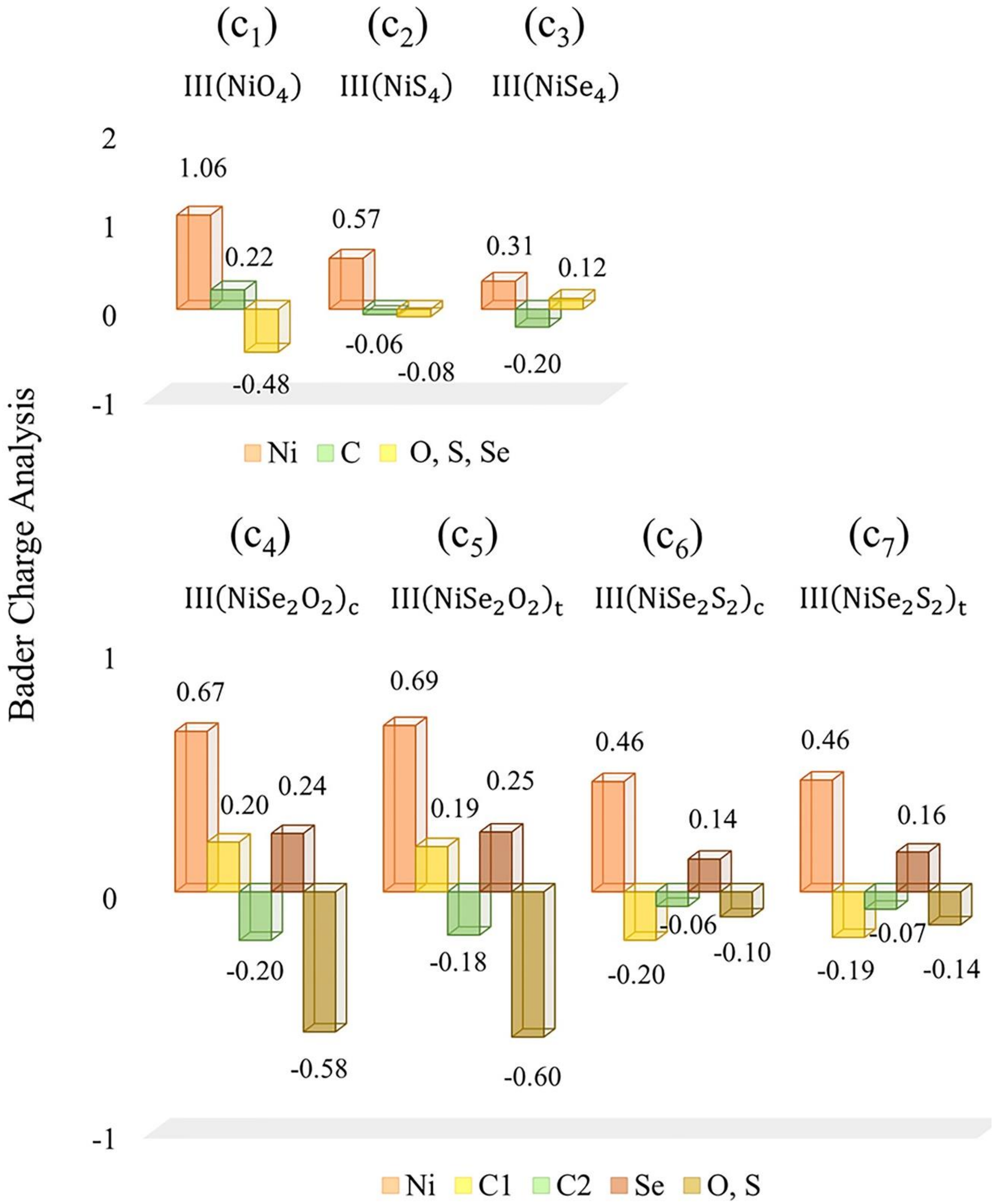
(c1) (c2) (c3)
III(NiO4) III(NiS4) III(NiSe4)
2
1.06
1
0.57
0.22
0.31
0.12
0
-0.06
-0.08
-0.20
-0.48
-1
Ni C O, S, Se
Bader Charge Analysis
(c4) (c5) (c6) (c7)
III(NiSe2O2)c III(NiSe2O2)t III(NiSe2S2)c III(NiSe2S2)t
1
0.67
0.69
0.46
0.46
0.20
0.24
0.19
0.25
0.14
0.16
0
-0.06
-0.07
-0.20
-0.18
-0.20
-0.10
-0.19
-0.14
-0.58
-0.60
-1
Ni C1 C2 Se O, S

Figure S7. Band structures of the twenty-one configurations. These include: $I(NiO_4)$, $I(NiS_4)$, $I(NiSe_4)$, $I(NiO_2S_2)_c$, $I(NiO_2S_2)_t$, $I(NiO_2Se_2)_c$, $I(NiO_2Se_2)_t$ and $II(NiO_4)$, $II(NiS_4)$, $II(NiSe_4)$, $II(NiS_2O_2)_c$, $II(NiS_2O_2)_t$, $II(NiS_2Se_2)_c$, $II(NiS_2Se_2)_t$ and $III(NiO_4)$, $III(NiS_4)$, $III(NiSe_4)$, $III(NiSe_2O_2)_c$, $III(NiSe_2O_2)_t$, $III(NiSe_2S_2)_c$, $III(NiSe_2S_2)_t$ structures, respectively. Subscripts 'c' and 't' denote *cis*- and *trans*-like configurations, respectively. (a_1-a_3), (b_1-b_3) and (c_1-c_3) represent the dispersion relations for homogeneous structures. The purple bands correspond to the Kagome bands, while the blue bands represent the other bands. The figures in the second row are zoomed in on the Kagome bands and SOC gaps.

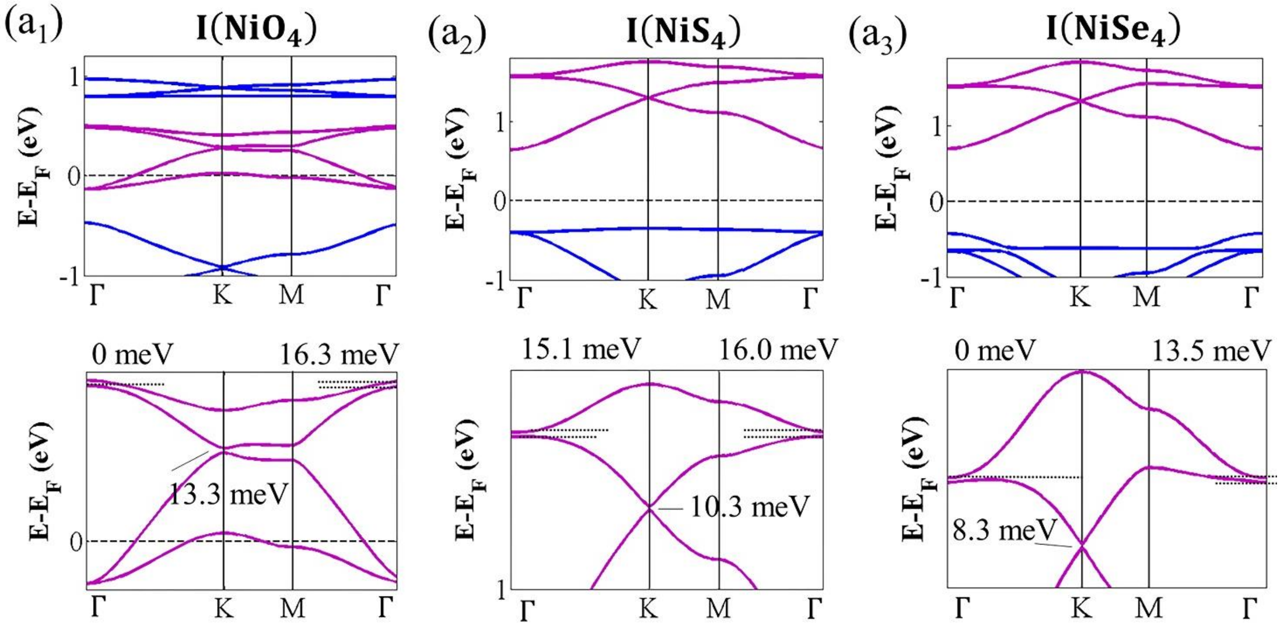

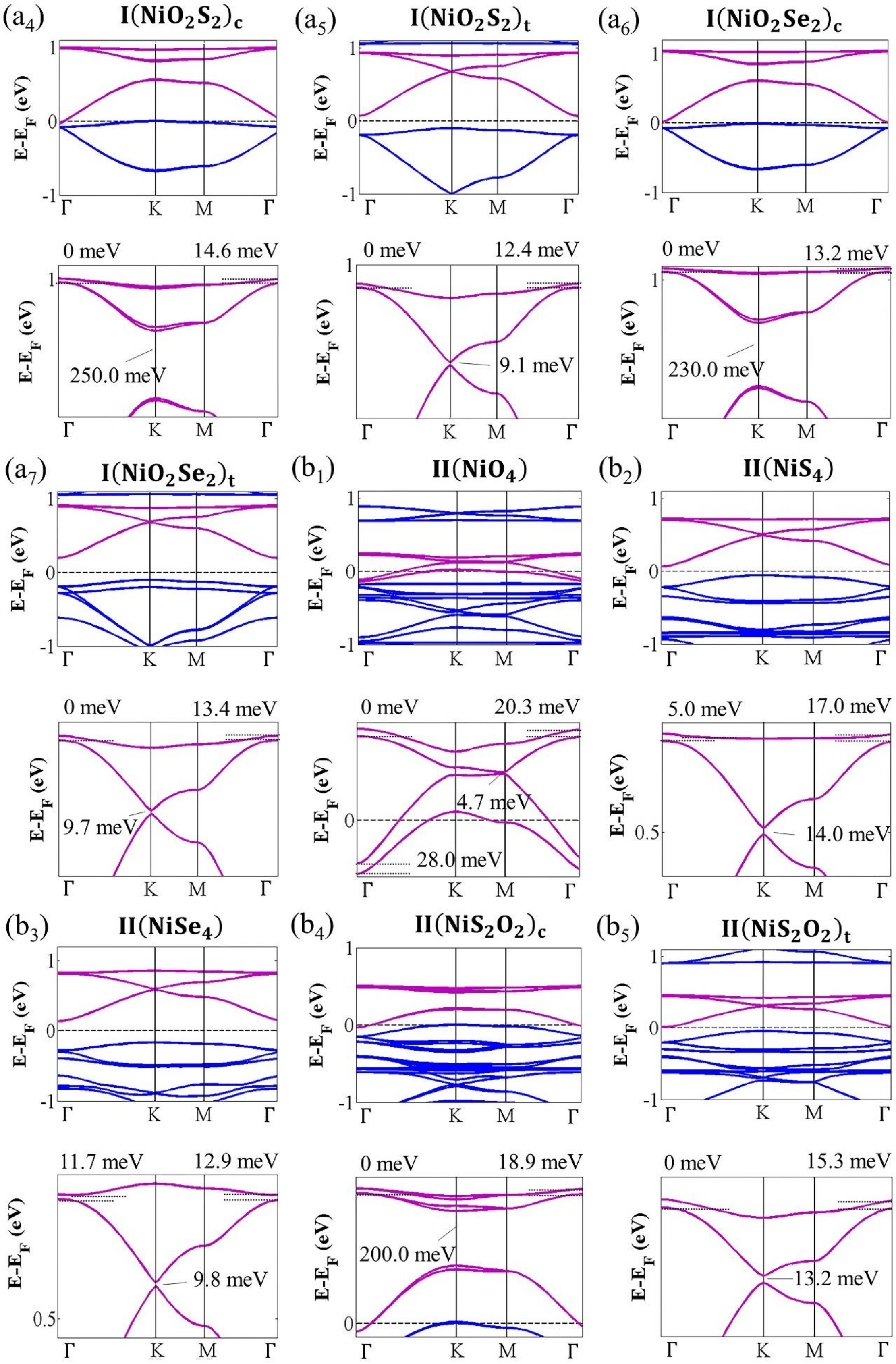
(a4)
I(NiO2S2)c
0 meV
14.6 meV
250.0 meV
(a5)
I(NiO2S2)t
0 meV
12.4 meV
9.1 meV
(a6)
I(NiO2Se2)c
0 meV
13.2 meV
230.0 meV
(a7)
I(NiO2Se2)t
0 meV
13.4 meV
9.7 meV
(b1)
II(NiO4)
0 meV
20.3 meV
4.7 meV
28.0 meV
(b2)
II(NiS4)
5.0 meV
17.0 meV
14.0 meV
(b3)
II(NiSe4)
11.7 meV
12.9 meV
9.8 meV
(b4)
II(NiS2O2)c
0 meV
18.9 meV
200.0 meV
(b5)
II(NiS2O2)t
0 meV
15.3 meV
13.2 meV
E-EF (eV)
Γ
K
M
Γ

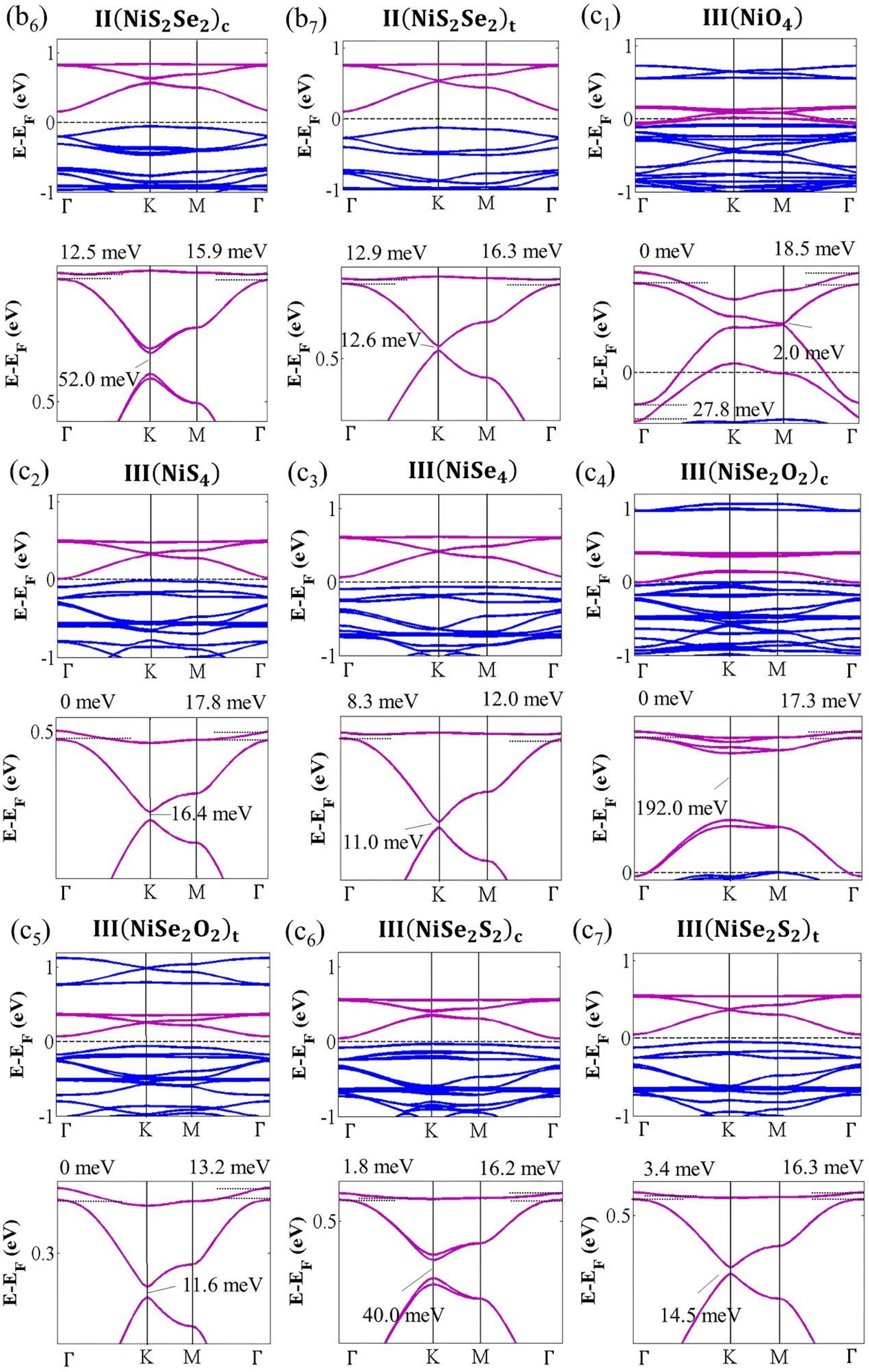
(b6) II(NiS2Se2)c
(b7) II(NiS2Se2)t
(c1) III(NiO4)
E-EF (eV)
Γ K M Γ
12.5 meV 15.9 meV
52.0 meV
12.9 meV 16.3 meV
12.6 meV
0 meV 18.5 meV
2.0 meV
27.8 meV
(c2) III(NiS4)
(c3) III(NiSe4)
(c4) III(NiSe2O2)c
0 meV 17.8 meV
16.4 meV
8.3 meV 12.0 meV
11.0 meV
0 meV 17.3 meV
192.0 meV
(c5) III(NiSe2O2)t
(c6) III(NiSe2S2)c
(c7) III(NiSe2S2)t
0 meV 13.2 meV
11.6 meV
1.8 meV 16.2 meV
40.0 meV
3.4 meV 16.3 meV
14.5 meV

Table S3. Dirac band gap without SOC (Wo-SOC/Dirac), Dirac band gap (Dirac), global band gap (global), and local band gap (local) for all twenty-one configurations. All energy values are given in meV. The Dirac gap refers to the band gap at the Dirac point $\boldsymbol{K}$; the global gap denotes the energy separation between the minimum of nearly flat band (FB) and the maximum of upper Dirac band across the BZ; and the local gap corresponds to the energy difference between the nearly FB and the upper Dirac band at the $\boldsymbol{\Gamma}$ point.

Structure	Wo-SOC/Dirac	Dirac	global	local
$\mathrm{I(NiO_4)}$	0.0	13.3	0.0	16.3
$\mathrm{I(NiS_4)}$	0.0	10.3	15.1	16.0
$\mathrm{I(NiSe_4)}$	0.0	8.3	0.0	13.5
$\mathrm{II(NiO_4)}$	0.0	4.7	0.0	20.3
$\mathrm{II(NiS_4)}$	0.0	14.0	5.0	17.0
$\mathrm{II(NiSe_4)}$	0.0	9.8	11.7	12.9
$\mathrm{III(NiO_4)}$	0.0	2.0	0.0	18.5
$\mathrm{III(NiS_4)}$	0.0	16.4	0.0	17.8
$\mathrm{III(NiSe_4)}$	0.0	11.0	8.3	12.0
$\mathrm{I(NiO_2S_2)_c}$	256.0	250.0	0.0	14.6
$\mathrm{I(NiO_2Se_2)_c}$	235.6	230.0	0.0	13.2
$\mathrm{II(NiS_2O_2)_c}$	224.0	200.0	0.0	18.9
$\mathrm{II(NiS_2Se_2)_c}$	64.0	52.0	12.5	15.9
$\mathrm{III(NiSe_2O_2)_c}$	209.6	192.0	0.0	17.3
$\mathrm{III(NiSe_2S_2)_c}$	59.6	40.0	0.0	16.2
$\mathrm{I(NiO_2S_2)_t}$	0.0	9.1	0.0	12.4
$\mathrm{I(NiO_2Se_2)_t}$	0.0	9.7	0.0	13.4
$\mathrm{II(NiS_2O_2)_t}$	0.0	13.2	0.0	15.3
$\mathrm{II(NiS_2Se_2)_t}$	0.0	12.6	12.9	16.3
$\mathrm{III(NiSe_2O_2)_t}$	0.0	11.6	0.0	13.2
$\mathrm{III(NiSe_2S_2)_t}$	0.0	14.4	3.6	16.2

Figure S8. Band structure of (a) pristine II(NiS_4), (b) II(NiS_4) doped with one electron, (c) II(NiS_4) doped with two electrons and (d) II(NiS_4) doped with three electrons and (e) II(NiS_4) doped with four electrons. The Fermi level shifts upward in all four cases.

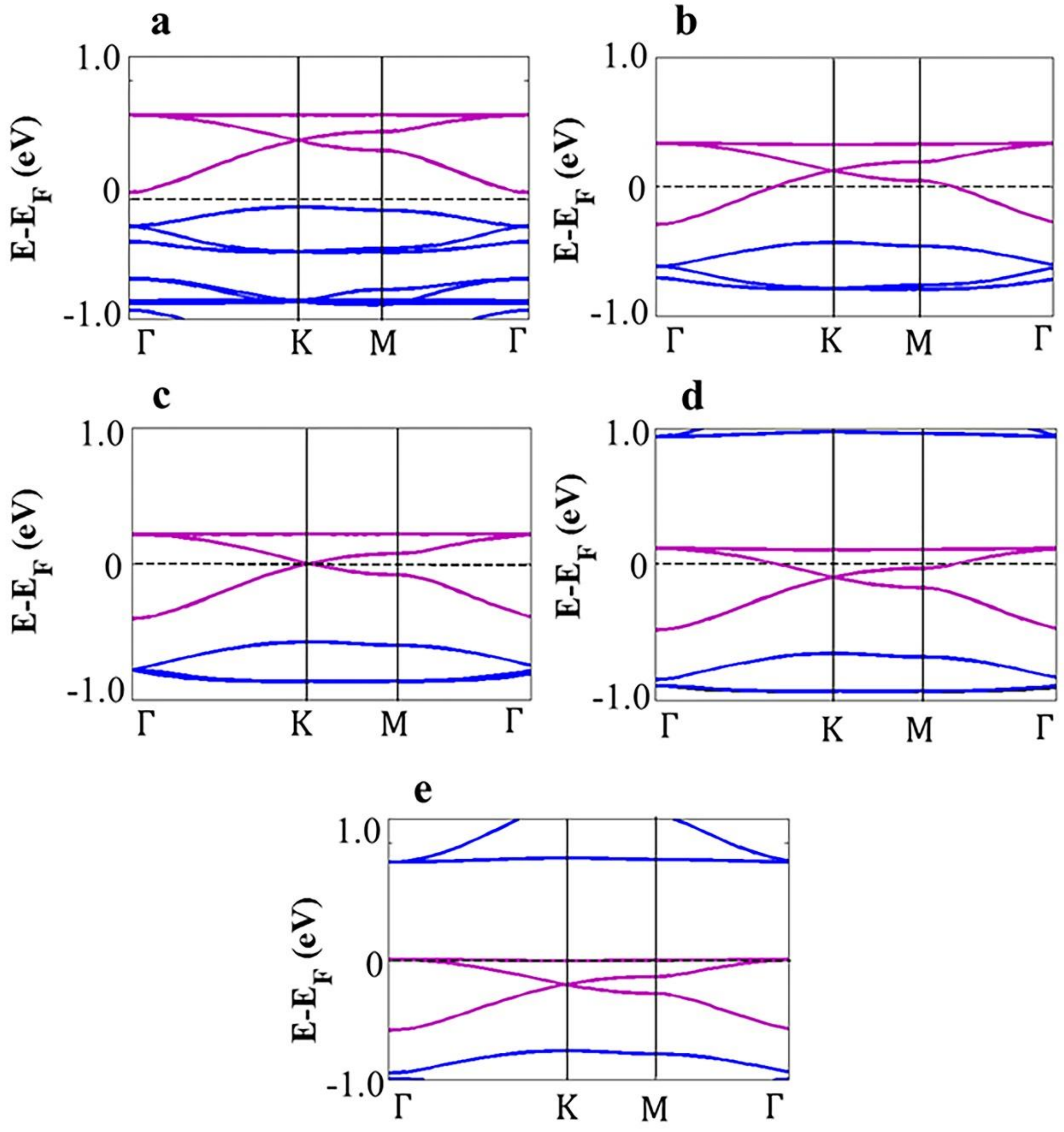

Table S4. Lattice parameter a (Å), Dirac band gap (Dirac), global band gap (global), and local band gap (local) for all twenty-one configurations under the two-electron doping concentration. All energy values are given in meV. The Dirac gap refers to the band gap at the Dirac point $\boldsymbol{K}$; the global gap denotes the energy separation between the minimum of the nearly flat band (FB) and the maximum of upper Dirac band across the BZ; and the local gap corresponds to the energy difference between the nearly FB and the upper Dirac band at the $\boldsymbol{\Gamma}$ point.

Structure	a (Å)	Dirac	global	local
$\mathrm{I(NiO_4)^{-2}}$	12.99	13.5	0.0	16.4
$\mathrm{I(NiS_4)^{-2}}$	12.99	10.3	12.9	15.6
$\mathrm{I(NiSe_4)^{-2}}$	12.99	8.4	0.0	13.4
$\mathrm{II(NiO_4)^{-2}}$	14.64	0.2	0.0	23.9
$\mathrm{II(NiS_4)^{-2}}$	14.64	15.4	8.5	18.6
$\mathrm{II(NiSe_4)^{-2}}$	14.64	11.3	13.6	14.6
$\mathrm{III(NiO_4)^{-2}}$	15.35	2.6	0.0	22.0
$\mathrm{III(NiS_4)^{-2}}$	15.35	19.1	0.0	20.2
$\mathrm{III(NiSe_4)^{-2}}$	15.35	13.5	10.0	14.4
$\mathrm{I(NiO_2S_2)_c^{-2}}$	12.99	59.5	0.0	14.1
$\mathrm{I(NiO_2Se_2)_c^{-2}}$	12.99	52.9	0.0	13.1
$\mathrm{II(NiS_2O_2)_c^{-2}}$	14.64	105.1	0.0	20.9
$\mathrm{II(NiS_2Se_2)_c^{-2}}$	14.64	17.0	14.1	17.6
$\mathrm{III(NiSe_2O_2)_c^{-2}}$	15.35	110.8	0.0	20.4
$\mathrm{III(NiSe_2S_2)_c^{-2}}$	15.35	15.6	2.6	18.5
$\mathrm{I(NiO_2S_2)_t^{-2}}$	12.99	9.5	0.0	12.8
$\mathrm{I(NiO_2Se_2)_t^{-2}}$	12.99	9.9	0.0	14.1
$\mathrm{II(NiS_2O_2)_t^{-2}}$	14.64	15.0	0.0	17.1
$\mathrm{II(NiS_2Se_2)_t^{-2}}$	14.64	13.8	14.3	17.6
$\mathrm{III(NiSe_2O_2)_t^{-2}}$	15.35	12.5	0.0	14.6
$\mathrm{III(NiSe_2S_2)_t^{-2}}$	15.35	16.7	4.2	18.4

Table S5. Cohesive energies (E_C) per atom (in eV) for pristine structures under two-electron doping concentration.

Structure	$I(NiO_4)^{-2}$	$II(NiS_4)^{-2}$	$III(NiSe_4)^{-2}$
E_C/atom (eV)	-7.59	-6.50	-6.14

Figure S9. Relativistic band structure with the projection of the spin operator $\hat{s}_z$ (color map) for the *cis*-like configurations, under two-electron doping concentration. The red and blue colors represent spin-up and spin-down states, respectively. The color scale indicates the expectation value $\langle\hat{s}_z\rangle$ of the spin operator $\hat{s}_z$ in units of $\hbar/2$. (a) Band structure of $\mathrm{I(NiO_2S_2)_c^{-2}}$, (b) $\mathrm{I(NiO_2Se_2)_c^{-2}}$, (c) $\mathrm{II(NiS_2O_2)_c^{-2}}$, (d) $\mathrm{II(NiS_2Se_2)_c^{-2}}$, (e) $\mathrm{III(NiSe_2O_2)_c^{-2}}$ and (f) $\mathrm{III(NiSe_2S_2)_c^{-2}}$.

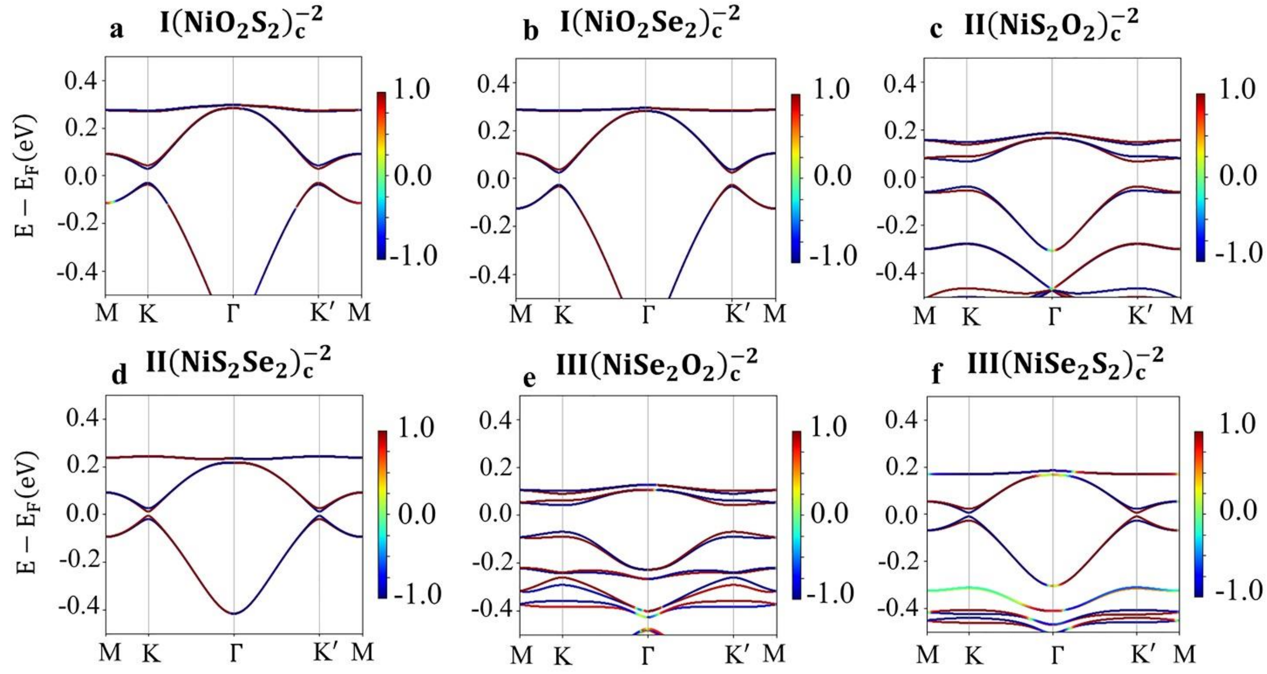

Figure S10. Planar electrostatic potential of pristine and *trans*-like configurations along the *x*- and *y*-axes. Panels show: (a) $\mathrm{I(NiO_4)}$, (b) $\mathrm{II(NiS_4)}$, (c) $\mathrm{III(NiSe_4)}$, (d$_1$) $\mathrm{I(NiO_2S_2)_t}$, (d$_2$) $\mathrm{I(NiO_2Se_2)_t}$, (d$_3$) $\mathrm{II(NiS_2O_2)_t}$, (d$_4$) $\mathrm{II(NiS_2Se_2)_t}$, (d$_5$) $\mathrm{III(NiSe_2O_2)_t}$ and (d$_6$) $\mathrm{III(NiSe_2S_2)_t}$.

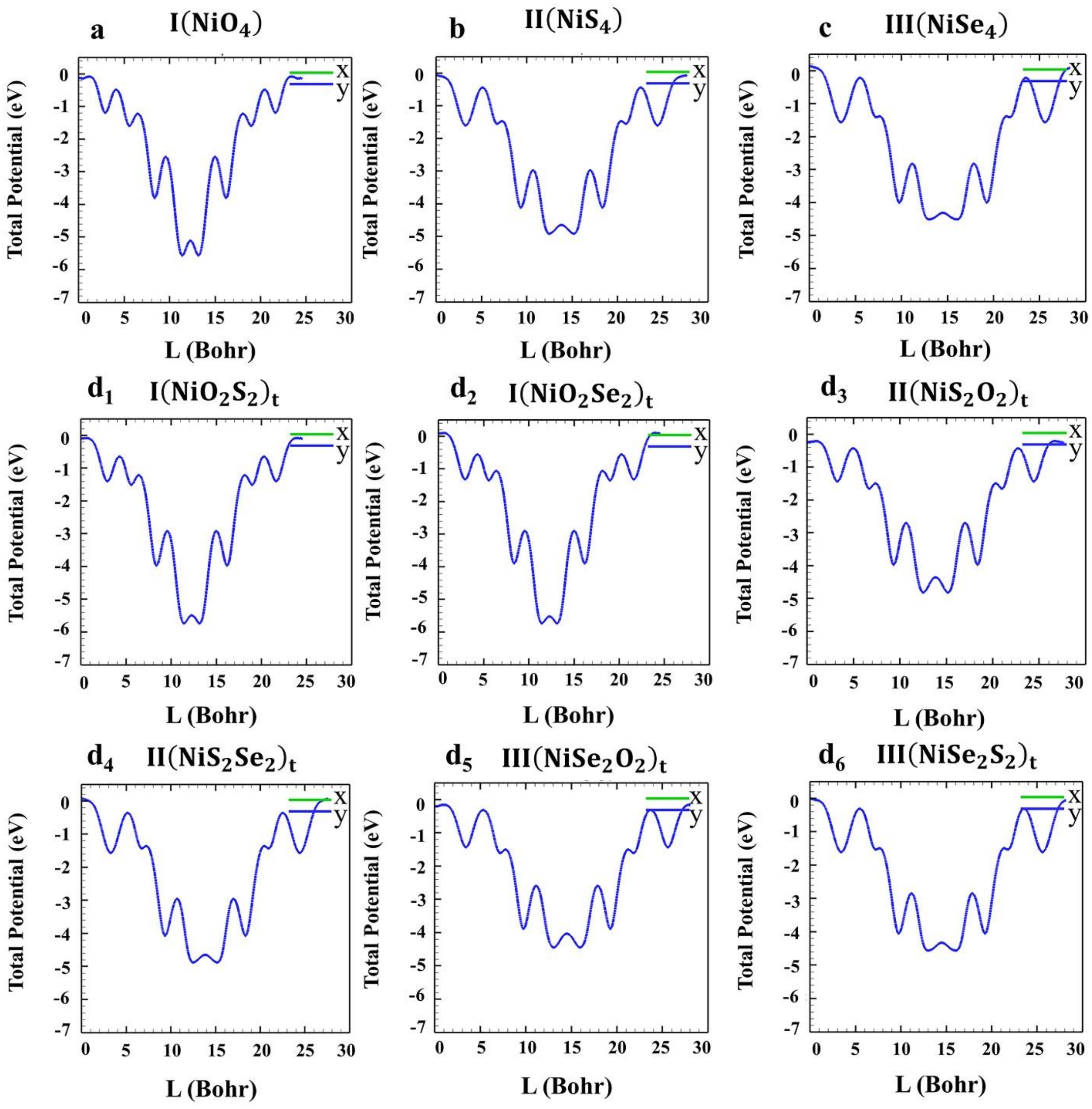

Figure S11. Valley-edge states of one-dimensional semi-infinite *cis*-like configurations. Panels (a_1-a_6) show the valley-edge states at the left edge for $\mathrm{I(NiO_2S_2)_c^{-2}}$, $\mathrm{I(NiO_2Se_2)_c^{-2}}$, $\mathrm{II(NiS_2O_2)_c^{-2}}$, $\mathrm{II(NiS_2Se_2)_c^{-2}}$, $\mathrm{III(NiSe_2O_2)_c^{-2}}$ and $\mathrm{III(NiSe_2S_2)_c^{-2}}$, respectively. Panels ($b_1$-$b_6$) display the corresponding valley edge states at the right edge. Red bands indicate the conducting edge states. Panels (c_1-c_6) present the band structures of one-dimensional nanoribbons with *cis*-like configurations, each with a width of 40 times the unit cell length. Gray bands represent bulk states (40 bands), while blue and red bands correspond to valley-edge states at the left and right edges, respectively. These panels effectively combine the information from the corresponding **a** and **b** panels. Panels (d_1-d_6) illustrate the BC distributions of the VB in the (k_x, k_y) plane.

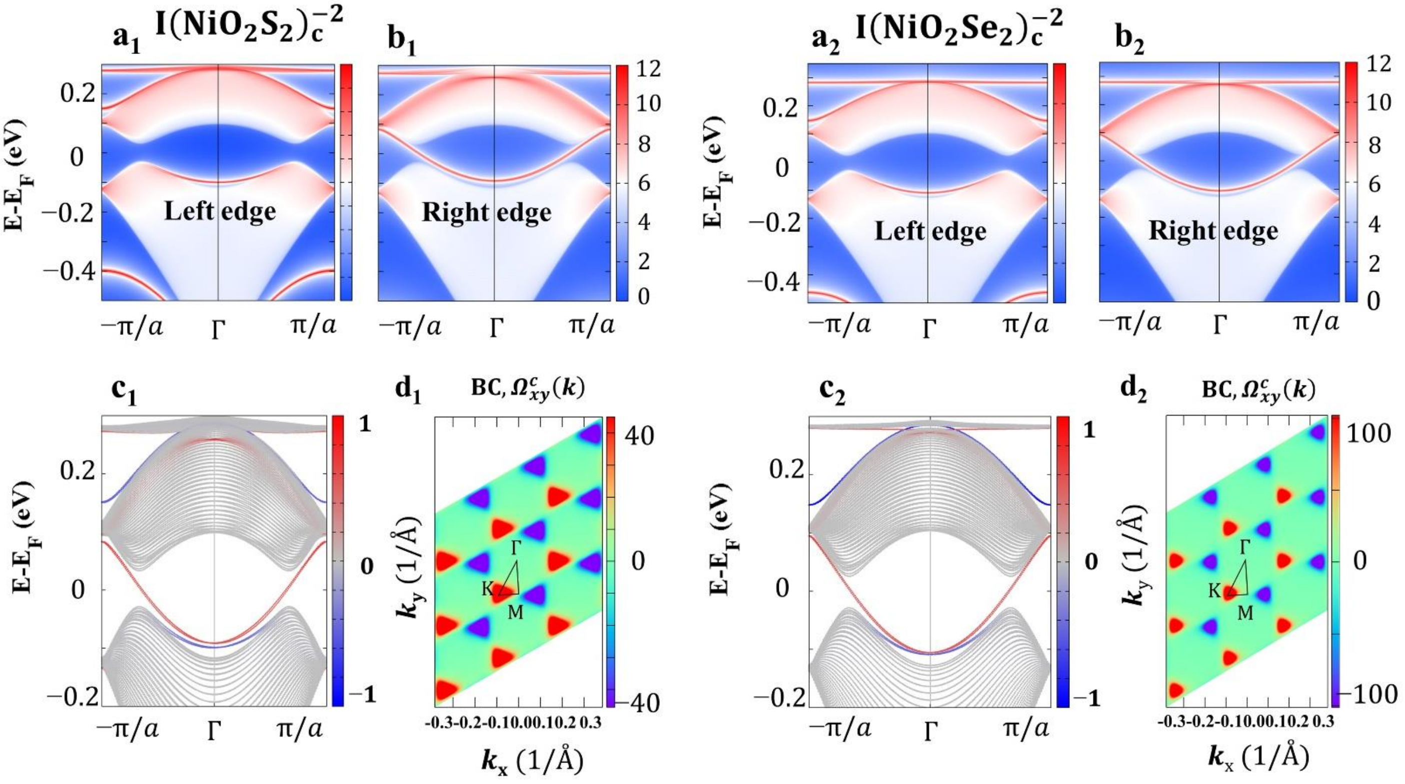

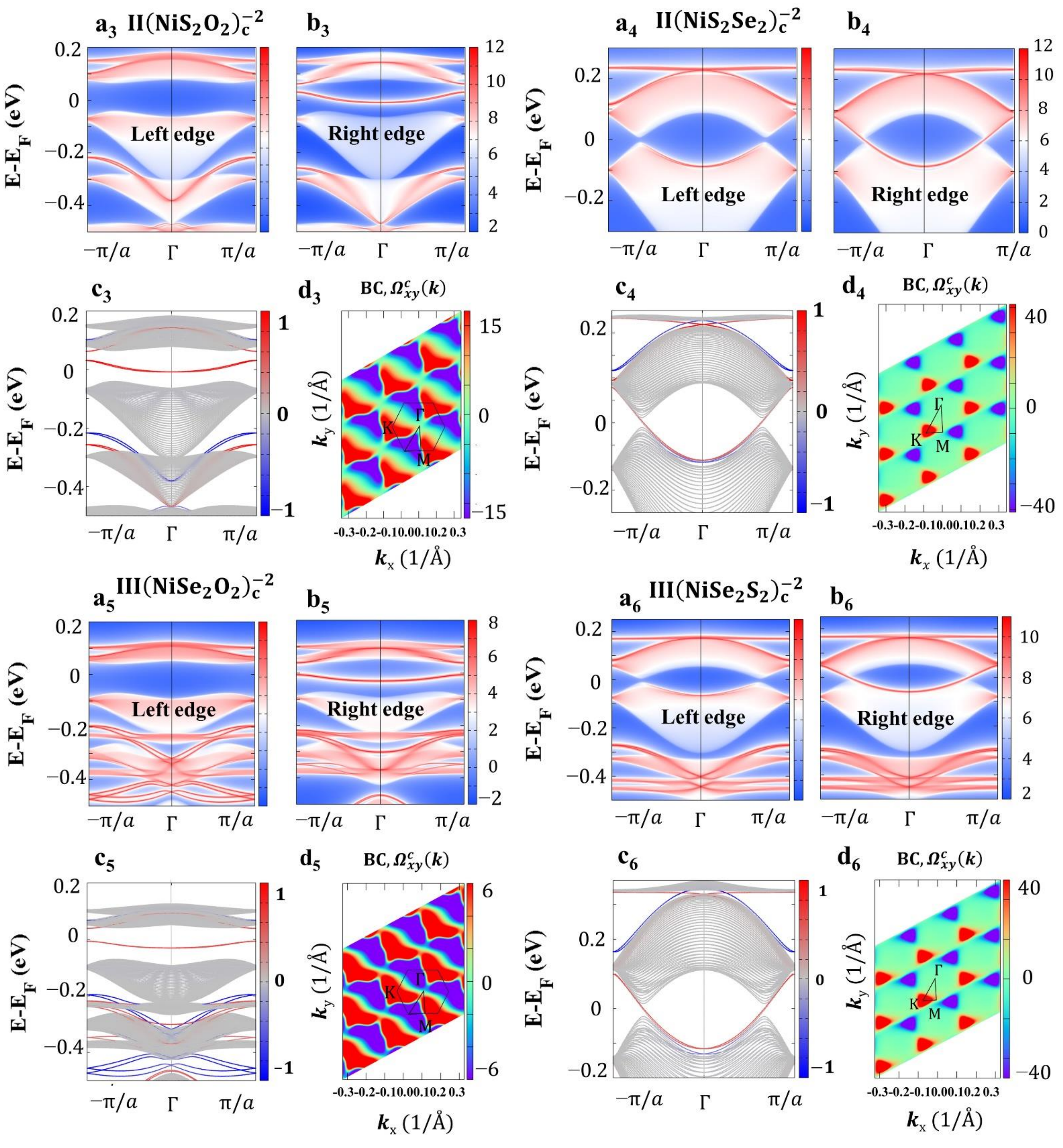

a3 II(NiS2O2)c−2
b3
Left edge
Right edge
E-EF (eV)
−π/a
Γ
π/a
a4 II(NiS2Se2)c−2
b4
c3
d3
BC, Ωcxy(k)
ky (1/Å)
kx (1/Å)
K
M
c4
d4
a5 III(NiSe2O2)c−2
b5
a6 III(NiSe2S2)c−2
b6
c5
d5
c6
d6

Figure S12. Edge configurations for $II(NiS_2Se_2)_c^{-2}$ are schematically illustrated, highlighting two distinct terminations. (a) Depicts a selenium-terminated edge on the left, while (b) shows a selenium-terminated edge on the right. The green and yellow circles are Se and S atoms, respectively. The corresponding edge states for each configuration are displayed.

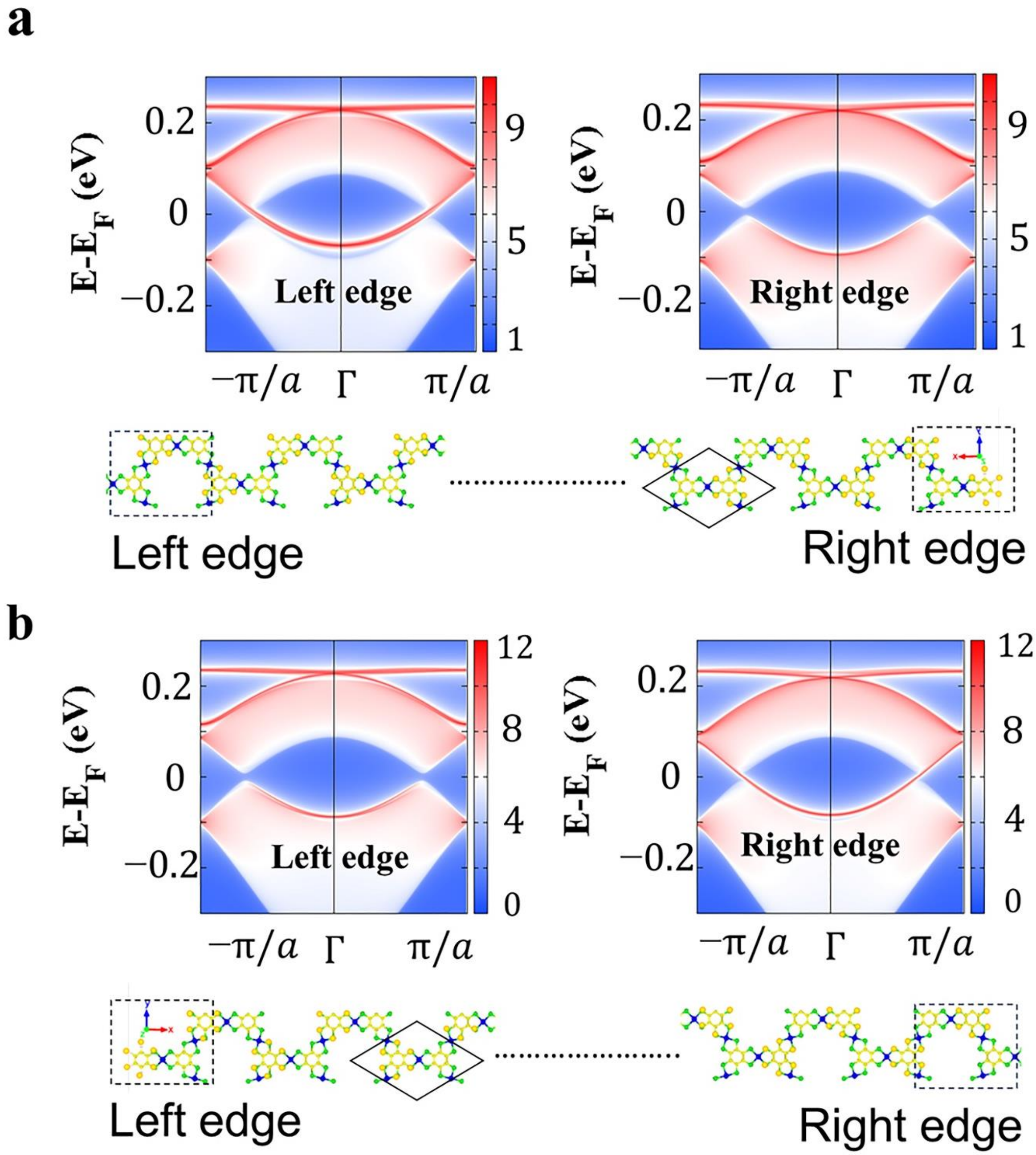

Figure S13. Schematic view of the nanoribbon geometry for the $I(NiO_2Se_2)_c^{-2}$, highlighting the edge atoms at both the left and right terminations. The nanoribbon has a width (W) of 52 *nm*, corresponding to approximately 40 times the lattice constant (12.99 Å). The corresponding nanoribbon band structure displays 40 gray bands representing bulk states, while red and blue bands indicate edge states localized at the right and left edges, respectively.

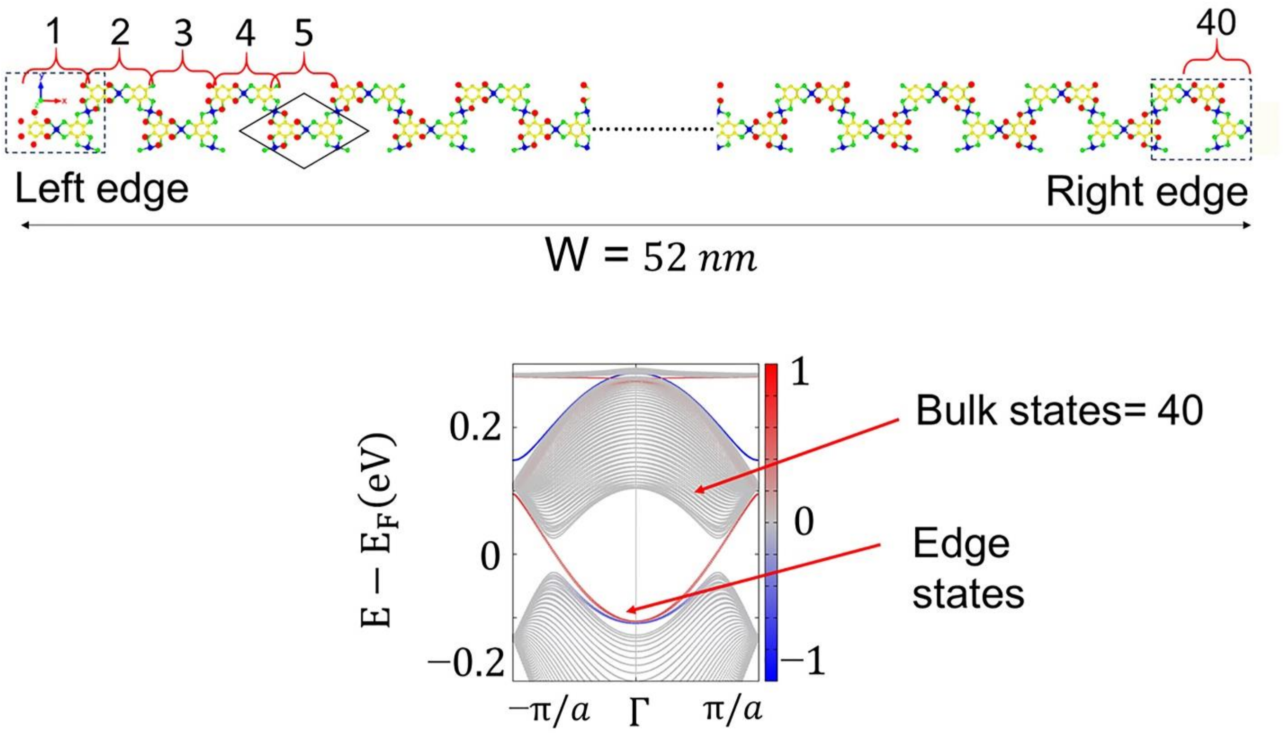

Figure S14. Ordinary BC of the VB in *cis*-like configurations of I, II and III$(NiX_2Y_2)_c^{-2}$ structures, calculated along high-symmetry lines without considering SOC. Panels show: (a) I$(NiO_2S_2)_c^{-2}$, (b) I$(NiO_2Se_2)_c^{-2}$, (c) II$(NiS_2O_2)_c^{-2}$, (d) II$(NiS_2Se_2)_c^{-2}$, (e) III$(NiSe_2O_2)_c^{-2}$ and (f) III$(NiSe_2S_2)_c^{-2}$.

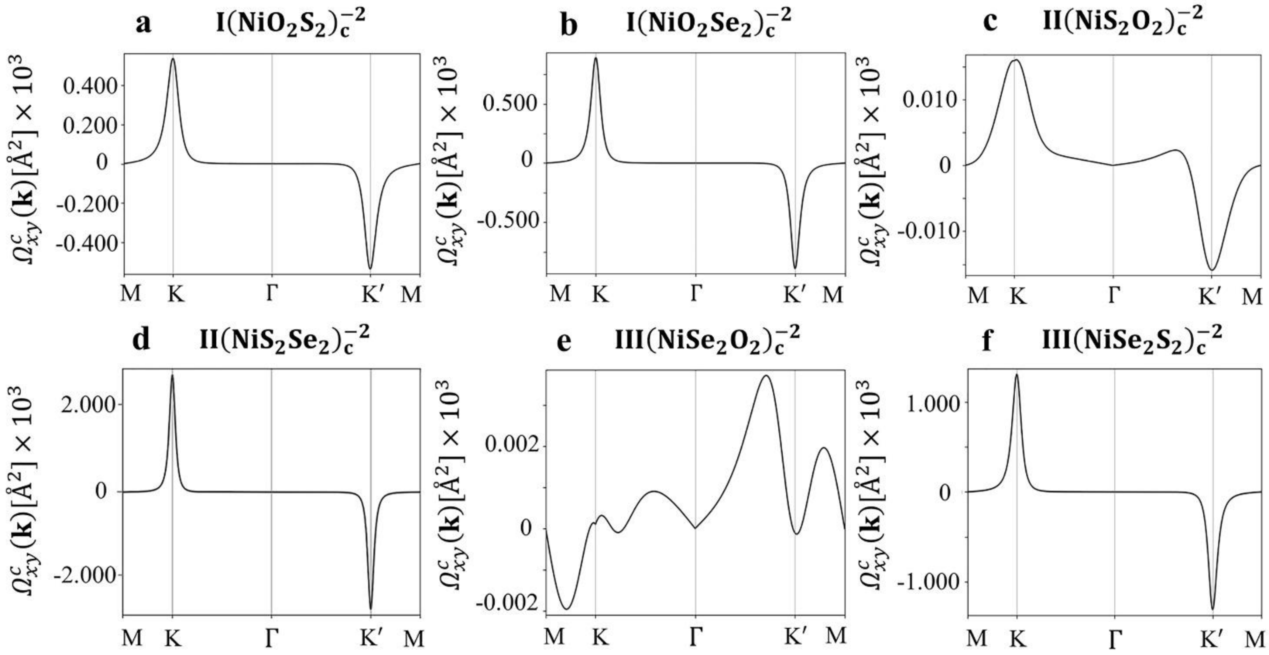

Figure S15. Planar electrostatic potential of two *trans*-like configurations, $II(NiS_2Se_2)_t^{-2}$ and $III(NiSe_2S_2)_t^{-2}$, along the *x* axes. (b) the corresponding charge density.

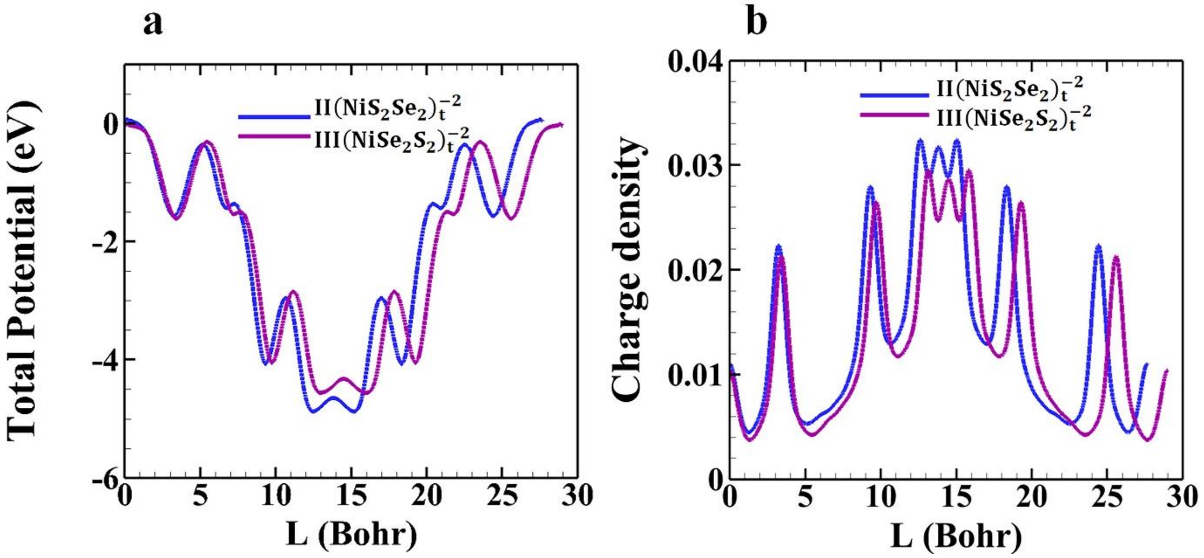

Figure S16. Helical edge states of 1D semi-infinite *trans*-like configurations. Panels (a_1-a_6) display the helical edge states at the left edge for $\mathrm{I(NiO_2S_2)_t^{-2}}$, $\mathrm{I(NiO_2Se_2)_t^{-2}}$, $\mathrm{II(NiS_2O_2)_t^{-2}}$, $\mathrm{II(NiS_2Se_2)_t^{-2}}$, $\mathrm{III(NiSe_2O_2)_t^{-2}}$ and $\mathrm{III(NiSe_2S_2)_t^{-2}}$, respectively. Panels ($b_1$-$b_6$) show the corresponding edge states at the right edge. Red bands indicate conducting edge states. Panels (c_1-c_6) present the band structures of 1D nanoribbons for the *trans*-like configurations, each with a width equivalent to 40 times the unit cell length. Gray bands represent bulk states (40 bands), while blue and red bands correspond to conducting helical edge states localized at the left and right edges, respectively. These "c" panels effectively combine the information from the corresponding **a** and **b** panels. Panels (d_1-d_6) illustrate the band-projected SBC, $\Omega^z_{n,xy}(\boldsymbol{k})$, in the vicinity of the Fermi level for the *trans*-like configurations. The energy axis is referenced to the Fermi level, indicated by the gray horizontal line. The color scale encodes the sign and magnitude of the SBC, defined as sgn $(\Omega^z_{n,xy}(\boldsymbol{k}))$ log $|\Omega^z_{n,xy}(\boldsymbol{k})|$.

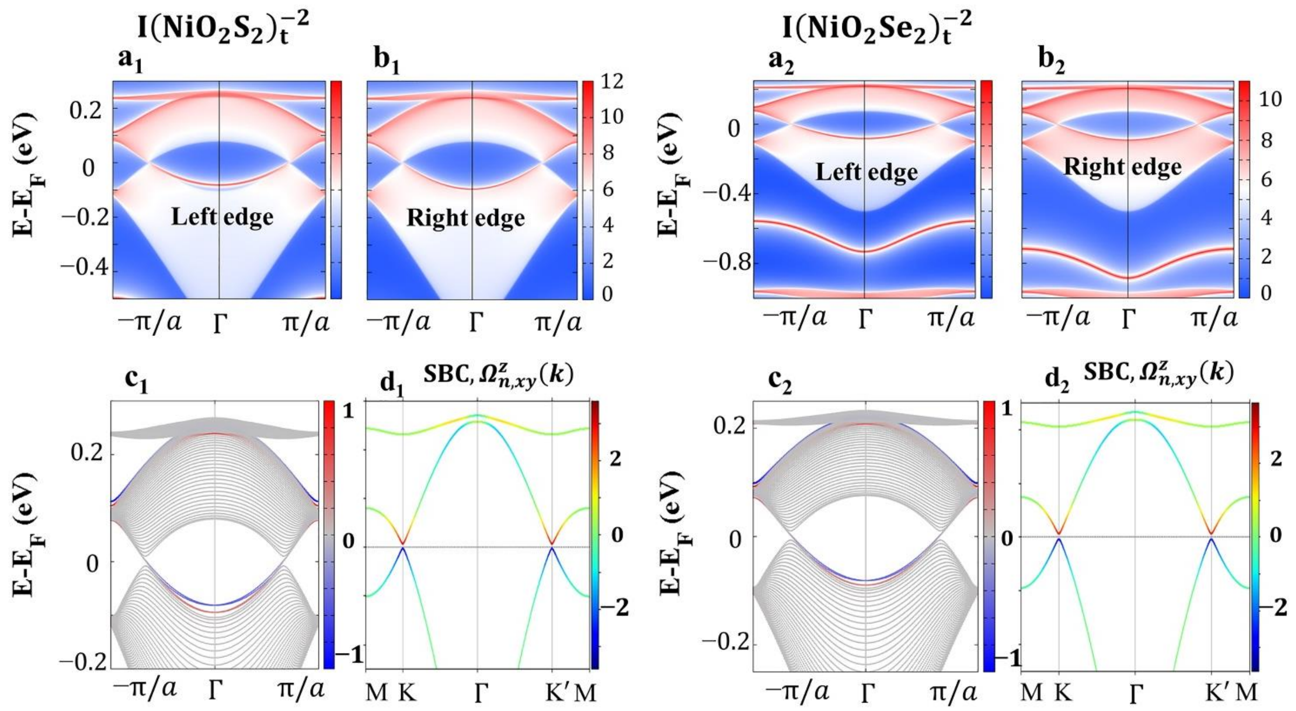

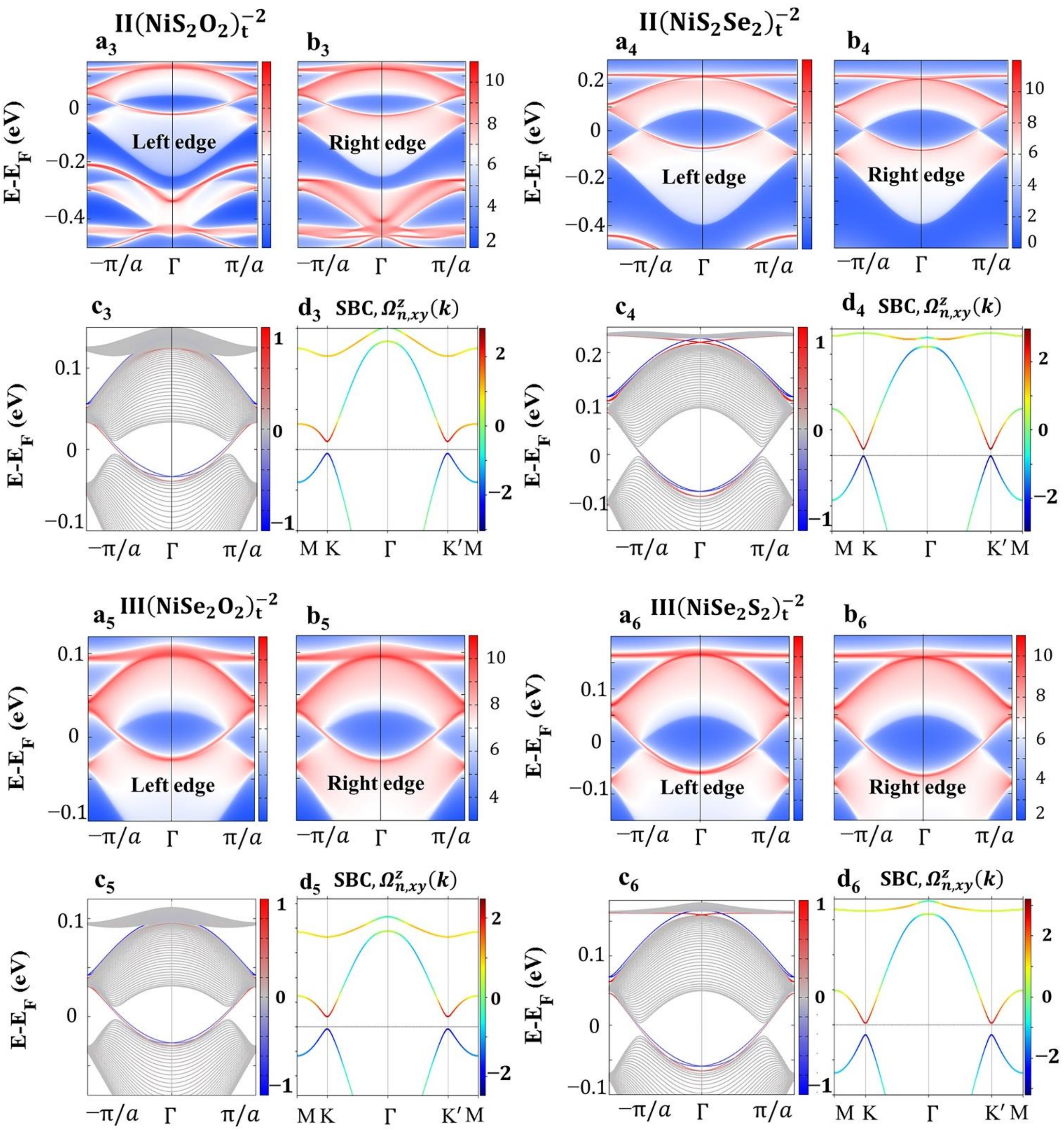

$II(NiS_2O_2)_t^{-2}$
a_3
b_3
Left edge
Right edge
$II(NiS_2Se_2)_t^{-2}$
a_4
b_4
E-E_F (eV)
$-\pi/a$
Γ
π/a
c_3
d_3 SBC, $\Omega^z_{n,xy}(k)$
c_4
d_4 SBC, $\Omega^z_{n,xy}(k)$
M K Γ K′ M
a_5 $III(NiSe_2O_2)_t^{-2}$
b_5
a_6 $III(NiSe_2S_2)_t^{-2}$
b_6
c_5
d_5 SBC, $\Omega^z_{n,xy}(k)$
c_6
d_6 SBC, $\Omega^z_{n,xy}(k)$

Figure S17. Edge states of 1D semi-infinite nonpristine homogeneous structures I, II and III(NiX_2Y_2), with X = Y = O, S or Se. Panels (a_1-a_6) show the calculated edge states at the left edge for I(NiS_4), I($NiSe_4$), II(NiO_4), II($NiSe_4$), III(NiO_4) and III(NiS_4), respectively. Panels (b_1-b_6) illustrate the corresponding edge states at the right edge. Red bands indicate conducting edge states. Panels (c_1-c_6) present the band structures of 1D nanoribbons for these configurations with the width of 40 times their unit cell length. Gray bands represent bulk states (40 bands), the blue and red bands correspond to the conducting edge states localized at the left and right edges, respectively. Panels (d_1-d_6) display the band-projected SBC, $\Omega^z_{n,xy}(\boldsymbol{k})$, near the Fermi level. The energy axis is relative to the Fermi level, indicated by the gray horizontal line. The color scale of the bands encodes the sign and magnitude of the SBC, defined as sgn ($\Omega^z_{n,xy}(\boldsymbol{k})$) log |$\Omega^z_{n,xy}(\boldsymbol{k})$|.

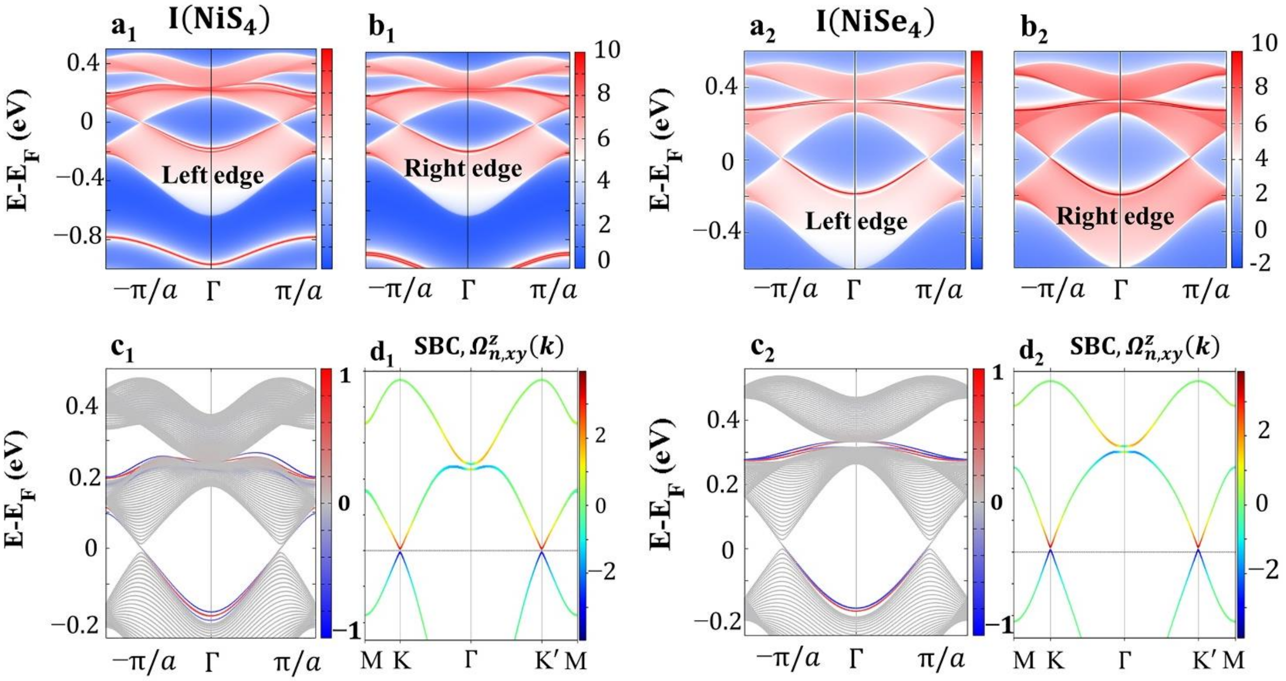

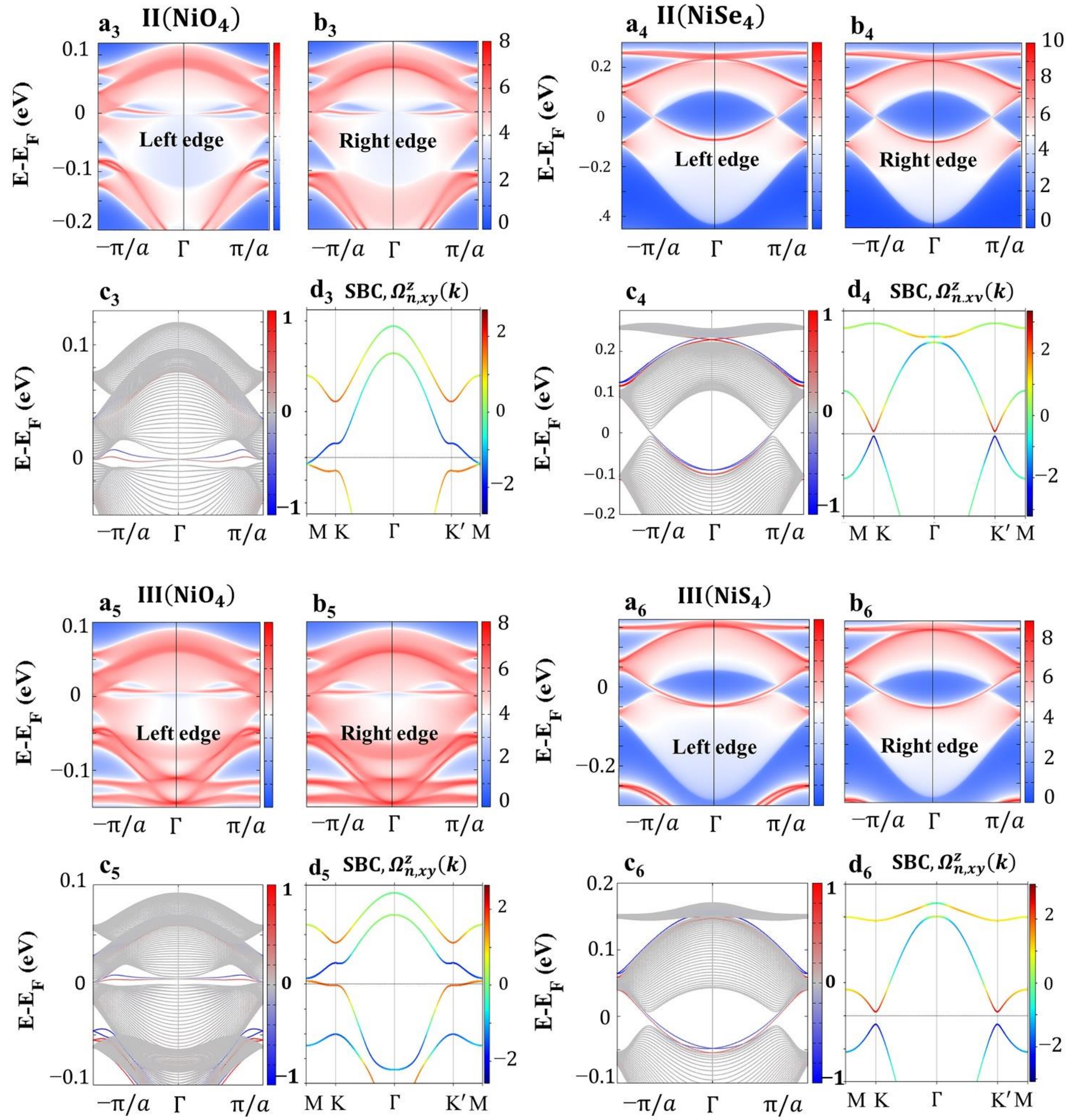
a3 II(NiO4)
b3
Left edge
Right edge
E-EF (eV)
−π/a Γ π/a
c3
d3 SBC, Ω^z_{n,xy}(k)
M K Γ K′ M
a4 II(NiSe4)
b4
c4
d4 SBC, Ω^z_{n,xy}(k)
a5 III(NiO4)
b5
c5
d5 SBC, Ω^z_{n,xy}(k)
a6 III(NiS4)
b6
c6
d6 SBC, Ω^z_{n,xy}(k)
